# CD1 and iNKT cells mediate immune responses against the GBS hemolytic lipid toxin induced by a non-toxic analog

**DOI:** 10.1371/journal.ppat.1011490

**Published:** 2023-06-29

**Authors:** Anna Furuta, Michelle Coleman, Raquel Casares, Ravin Seepersaud, Austyn Orvis, Alyssa Brokaw, Phoenicia Quach, Shayla Nguyen, Erin Sweeney, Kavita Sharma, Grace Wallen, Rhea Sanghavi, Jaime Mateos-Gil, Juan Manuel Cuerva, Alba Millán, Lakshmi Rajagopal

**Affiliations:** 1 Center for Global Infectious Disease Research, Seattle Children’s Research Institute, Seattle, Washington, United States of America; 2 Department of Global Health, University of Washington, Seattle, Washington, United States of America; 3 Department of Organic Chemistry, University of Granada, Granada, Spain; 4 Department of Pediatrics, University of Washington, Seattle, Washington, United States of America; Lunds universitet Medicinska fakulteten, SWEDEN

## Abstract

Although hemolytic lipids have been discovered from many human pathogens including Group B Streptococcus (GBS), strategies that neutralize their function are lacking. GBS is a leading cause of pregnancy-associated neonatal infections, and adult GBS infections are on the rise. The GBS hemolytic lipid toxin or granadaene, is cytotoxic to many immune cells including T and B cells. We previously showed that mice immunized with a synthetic nontoxic analog of granadaene known as **R-P4** had reduced bacterial dissemination during systemic infection. However, mechanisms important for **R-P4** mediated immune protection was not understood. Here, we show that immune serum from **R-P4**-immunized mice facilitate GBS opsonophagocytic killing and protect naïve mice from GBS infection. Further, CD4^+^ T cells isolated from **R-P4**-immunized mice proliferated in response to **R-P4** stimulation in a CD1d- and iNKT cell-dependent manner. Consistent with these observations, **R-P4** immunized mice lacking CD1d or CD1d-restricted iNKT cells exhibit elevated bacterial burden. Additionally, adoptive transfer of iNKT cells from **R-P4** vaccinated mice significantly reduced GBS dissemination compared to adjuvant controls. Finally, maternal **R-P4** vaccination provided protection against ascending GBS infection during pregnancy. These findings are relevant in the development of therapeutic strategies targeting lipid cytotoxins.

## Author summary

Although many pathogens encode lipid toxins that contribute to disease pathogenesis, strategies to neutralize these toxins are lacking. The hemolytic lipid toxin encoded by Group B Streptococcus (GBS) is a major virulence factor that promotes disease including preterm births, in utero fetal injury and morbidity of human neonates and adults. No strategies exist to mitigate the effects of the GBS toxin, and neutralizing antibodies have not been isolated from infected patients. The global burden of GBS disease remains high with approximately 4 million neonatal deaths occurring worldwide each year and increasing rates of infections occur in adults. Using chemical synthesis, we synthesized a derivative of GBS toxin known as **R-P4**. We show that vaccination with this non-toxic analog induces the production of antibodies that promotes GBS killing, confers protection to naïve mice, neutralizes the cytotoxic activity and diminishes GBS infection during pregnancy. Furthermore, we show that lipid analog mediated protection requires antigen presentation by host molecules such as CD1d and immune responses of CD1d restricted T cells such as iNKT cells. These results provide the foundation for the design of novel therapeutic strategies that target lipid cytotoxins.

Despite observations that lipid toxins continue to increase the severity and rate of infections caused by pathogens such as GBS, Mycobacteria and Pseudomonas, strategies to neutralize these toxins remain undefined. Our results indicate that a nontoxic analog of one such toxin can induce protective immunity during systemic and pregnancy associated infection, through lipid antigen presentation which provides the foundation for promising future strategies to neutralize the effect of lipid toxins during human infections.

## Introduction

Several bacterial pathogens produce cytotoxins that impair host defenses by breaching cellular barriers and killing innate and adaptive immune cells [1–5]. While many bacterial toxins are proteinaceous in nature [6,7], some bacterial toxins are lipids [8]. Although remarkable progress has been made to define the function of protein toxins and to derive toxoid variants for disease attenuation [9–13], these advances are substantially lacking for bacterial lipid toxins. Difficulties encountered with purification and solubility of lipid toxins and with modifying lipid structures to identify key elements important for cytotoxic function have posed significant challenges. Examples of bacterial lipid toxins include the rhamnolipids of *Pseudomonas aeruginosa* [14,15], mycolactone of *Mycobacterium ulcerans* [16,17], and hemolysin of Group B *Streptococcus* (GBS, [18–21]).

GBS has been recognized as a leading cause of neonatal disease [22]. Invasive GBS disease in neonates is typically associated with pneumonia, sepsis and in severe cases, meningitis [23–26]. Rates of neonatal GBS disease remain high, with 300,000 neonatal disease cases and at least 90,000 infant deaths each year [22]. GBS is also a pathogen in nonpregnant adults, especially those with advanced age or underlying health conditions [27–29]. In fact, rates of invasive GBS infection in nonpregnant adults continue to rise [28–30]. Invasive GBS disease in nonpregnant adults is typically associated with skin or soft-tissue infections or bacteremia, often requiring hospitalization and intensive care management [29]. Currently, there is no FDA-approved vaccine for GBS and the sole therapeutic strategy for neonates and adults is antibiotic treatment [31–34]. However, the emergence of antibiotic resistant GBS isolates is concerning [31] and thus, the development of a GBS vaccine is a global health priority.

**Fig 1:**
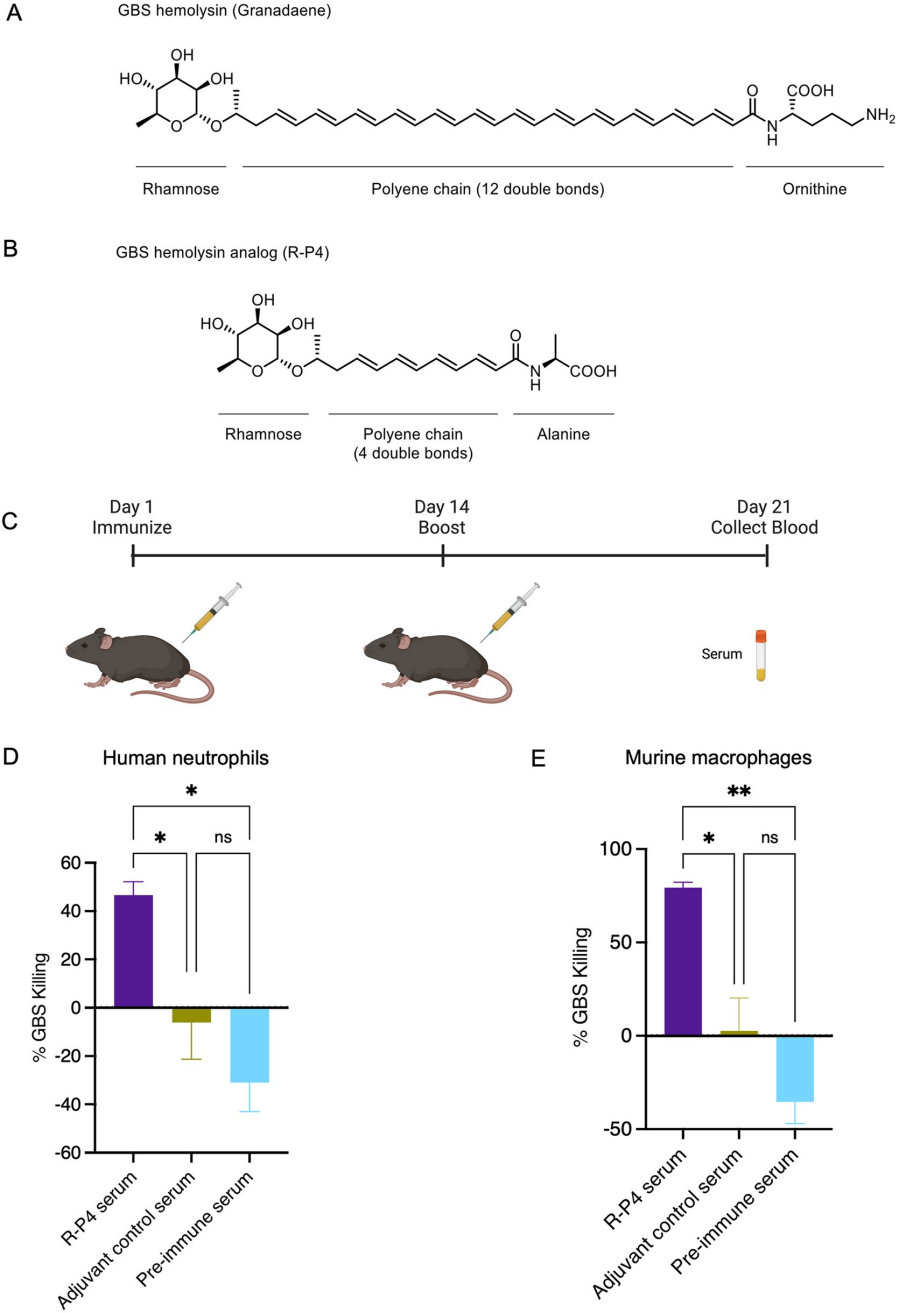
Immunization with R-P4 induces antibodies that mediate GBS opsonophagocytic killing *in vitro*. (A) Structure of GBS hemolysin or Granadaene which contains a rhamnose sugar, a polyene chain with 12 double bonds, and a terminal ornithine amino acid; generated using the software ChemDraw. (B) Structure of **R-P4**, which is composed of a rhamnose sugar, a polyene chain with 4 double bonds, and a terminal alanine; generated using the software ChemDraw. (C) Schematic created using BioRender.com displaying the experimental design wherein mice were immunized intraperitoneally (i.p). with an emulsion of **R-P4** and complete Freund’s adjuvant and boosted 14 days later with an emulsion of **R-P4** and incomplete Freund’s adjuvant. After 21 days, serum was collected for opsonophagocytosis assay. (D) Opsonophagocytic killing of HH GBS (WT NCTC10/84) by human neutrophils in the presence of **R-P4** immune serum. HH GBS was pre-treated with inactivated serum from either **R-P4** immunized or adjuvant-treated mice or with inactivated pre-immune serum for 30 minutes and then incubated with neutrophils for 60 minutes. Surviving CFU were enumerated by dilution plating onto TSA and percent killing of GBS was calculated. Treatment groups were compared using a one-way ANOVA test with Tukey’s post-test. Mean and SEM from at least three independent experiments performed in triplicate are shown. *Indicates p < 0.05, ns indicates not significant, or p ≥ 0.05. (E) Opsonophagocytic killing of HH GBS (WT NCTC10/84) by mouse macrophages in the presence of **R-P4** immune serum. HH GBS was opsonized with inactivated serum from **R-P4** immunized or adjuvant-treated mice or with inactivated pre-immune serum and then incubated with macrophages for 60 minutes. Surviving CFU were enumerated by dilution plating onto TSA and percent killing of GBS was calculated. Treatment groups were compared using a one-way ANOVA test with Tukey’s post-test. Mean and SEM are shown from three independent samples performed in duplicate are shown. ** Indicates p < 0.01, *indicates p < 0.05, ns indicates not significant or p ≥ 0.05.

Hemolytic activity is a key determinant of GBS virulence and pathogenesis. Non-hemolytic GBS strains are usually significantly attenuated for virulence in various models of infection [35–38]. Hyperhemolytic GBS strains have also been isolated from cases of severe, invasive disease in nonpregnant adults, neonates, and women in preterm labor [39–42]. The GBS hemolysin (or the pigmented lipid known as granadaene) subverts host immune responses by inducing cytotoxicity in innate and adaptive immune cells, including mast cells, macrophages, neutrophils, T cells and B cells [18–20,43]. During natural infection, hemolysin-specific B cell and T cell responses have not been identified or characterized, likely due to its cytotoxicity to these cells [20]. Granadaene is a hemolytic ornithine rhamnolipid containing 12 double bonds in its polyene chain, and this polyene is flanked by a rhamnose sugar on one end and an ornithine amino acid at the other end (Fig 1A), [41,44]. Using a panel of granadaene analogs derived through chemical synthesis, we previously showed that the polyene chain length and the presence of terminal groups (i.e., ornithine or rhamnose) are essential for full hemolysis [20]. One analog, known as **R-P4**, contains a polyene chain with 4 double bonds and a terminal alanine amino acid (Fig 1B, also see [20]). The **R-P4** analog lacked the cytotoxic properties of granadaene and thus, was well tolerated by innate and adaptive immune cells [20]. Notably, immunization of adult mice with **R-P4** reduced bacterial dissemination following systemic challenge with hyperhemolytic GBS [20]. Despite these advances, the host mechanisms important for protection associated with **R-P4** immunization remain undefined.

In this study, we aimed to characterize the adaptive immune response to the **R-P4** lipid analog to better define protection and to inform vaccine strategies. In contrast to peptide antigens, lipid antigens can be presented to T cells through the non-polymorphic CD1 family of major histocompatibility complex (MHC) class I-like antigen-presenting molecules. While human antigen-presenting cells (*e*.*g*. dendritic cells) express four functionally non-redundant isotypes of CD1 (CD1a, CD1b, CD1c and CD1d) on their surface, only CD1d presents surface antigen to T cells in mice [45–49]. CD1d-restricted T cells are T cell populations that recognize antigens presented on CD1d. A CD1d-restricted T cell subset known as invariant NKT (iNKT) cells are thought to respond to lipid antigen stimulation [50,51]. iNKT cells have an invariant T cell receptor (TCR) with identical TCRα chains (TRAV_11_-TRAJ_18_ in mice, TRAV_10_-TRAJ_18_ in humans) paired to a restricted set of TCRβ chains [52–54]. Studies have shown that CD4 engagement by CD1d potentiates iNKT cell activation during lipid antigen presentation [55]. iNKT cells are important in controlling infection by producing protective cytokines and through the recruitment and activation of phagocytes as observed for *Mycobacterium tuberculosis* [56,57], *Borrelia burgdorferi* [58–61], and *Streptococcus pneumoniae* [62–64].

Given that the **R-P4** analog is a lipid antigen, we examined the role of CD1d and CD1d- restricted T cells in the adaptive immune response to **R-P4** immunization. Here, we demonstrate that antibodies generated in response to **R-P4** immunization facilitate clearance of GBS both *in vitro* and *in vivo*. Additionally, CD4^+^ T cells proliferated in response to **R-P4** stimulation in a CD1d-dependent manner. Further, we identified that iNKT cells are a critical T cell subset for generating protective response to GBS due to **R-P4** immunization. Finally, we show that **R-P4** provides protection against ascending GBS infection in pregnant mice. Together, these data provide novel insights into the protective adaptive immune responses from a nontoxic analog that mitigates the effects of a cytotoxic lipid critical to GBS virulence.

## Results

### Serum from R-P4 vaccinated mice increase GBS opsonophagocytic killing

We previously showed that immunization with **R-P4** diminished GBS infection and generated antibodies reactive to GBS pigment, granadaene [20]. Thus, we hypothesized that antibodies induced by **R-P4** immunization may facilitate GBS bacterial clearance through opsonophagocytic killing by neutrophils. To test this hypothesis, mice were first immunized intraperitoneally (i.p) with an emulsion of **R-P4** and complete Freund’s adjuvant and boosted 14 days later with **R-P4** and incomplete Freund’s adjuvant as described previously [20]. Control mice were immunized with only the adjuvant using the same immunization schedule (Fig 1C). On day 21 post immunization, serum was collected from the immunized and control mice, as described [20]. We then assessed if the immune serum from **R-P4**-vaccinated mice facilitated GBS opsonophagocytic killing by human neutrophils. To this end, hyperhemolytic (HH) GBS (WT serotype V NCTC10/84) was pre-treated with inactivated **R-P4** immune serum for 30 minutes, followed by incubation with human neutrophils and rabbit complement for 60 minutes. Controls included GBS pre-treated with adjuvant serum or pre-immune serum. Bacterial killing was measured by serial dilution and plating before and after incubation (for details, see *Material and Methods*). The results shown in Fig 1D indicate that HH GBS pre-treated with **R-P4** immune serum were more readily killed by neutrophils compared to the control groups.

To determine if similar observations of opsonophagocytic killing could be seen with mouse phagocytes, we performed opsonophagocytic killing assays using the RAW264.7 murine macrophage cell line as a source of phagocytic cells as described [65]. Briefly, WT GBS NCTC10/84 was opsonized by incubation with inactivated **R-P4** immune serum in the presence of rabbit complement for 60 min at 4°C. Controls included adjuvant and pre-immune sera. After opsonization, murine macrophages were added, and the samples were incubated at 37°C for 60 min. Bacterial killing was measured by serial dilution and plating before and after incubation. The results shown in Fig 1E indicates that HH GBS pre-treated with **R-P4** immune serum were more readily killed by murine macrophages compared to the control groups. Taken together, these data confirm that **R-P4** immunization facilitated GBS clearance by phagocytes such as neutrophils and macrophages.

### Serum of R-P4 vaccinated mice diminishes GBS infection in naïve mice

**Fig 2:**
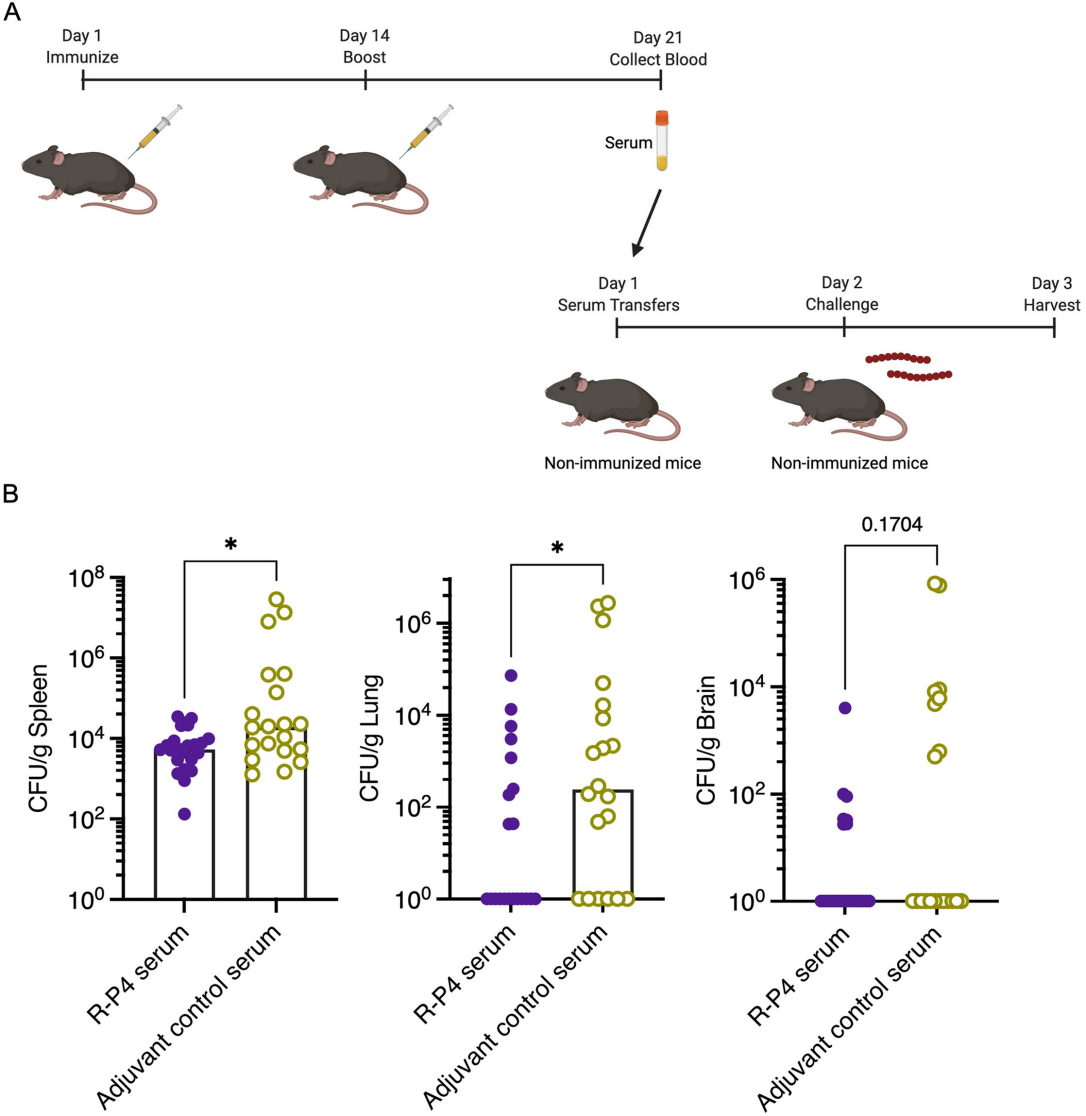
R-P4 immunization generates antibody responses that are protective *in vivo*. (A) Schematic created using BioRender.com displaying the serum transfer schedule. Mice were immunized i.p with an emulsion of **R-P4** and complete Freund’s adjuvant and boosted 14 days later with an emulsion of **R-P4** and incomplete Freund’s adjuvant. Adjuvant control mice received the adjuvant on the same schedule. After 21 days, blood was collected from the **R-P4** immunized or adjuvant-treated mice and serum from the blood was transferred intravenously (i.v) to naïve recipient mice, respectively. One day post serum transfer, the recipient mice were challenged i.p with HH GBS (WT NCTC10/84). (B) At 24 hours post GBS infection, mice were euthanized, and spleen, lungs and brains were collected to enumerate GBS CFU in each tissue. Sample size for each treatment group: *n = 23* for **R-P4** immune serum recipients and *n = 20* for adjuvant serum recipients. Data points represent individual mice, with the horizontal line representing the median. Mann-Whitney test was used to compare treatment groups. * Indicates p < 0.05.

To test if antibodies generated from **R-P4** immunization are protective *in vivo*, immune serum obtained from **R-P4**-immunized mice was administered to naive recipient mice intravenously (i.v). Controls included naïve recipient mice that received serum from adjuvant immunized mice. Approximately 24 hours after serum transfer, mice were challenged with HH GBS (1 x 10^7^ CFU, i.p, see Fig 2A for scheme). The recipient mice were euthanized the following day and lungs, spleen, and brain were collected. Tissues were homogenized and GBS CFU was enumerated using methods described [20]. Fig 2B shows that mice that received **R-P4** immune serum exhibited significantly reduced GBS bacterial burden in the lungs and spleen (with trends towards lower infection in the brain), compared to control mice. Collectively, these data suggest that **R-P4** immunization generates protective antibody responses *in vitro* and *in vivo*.

**Fig 3:**
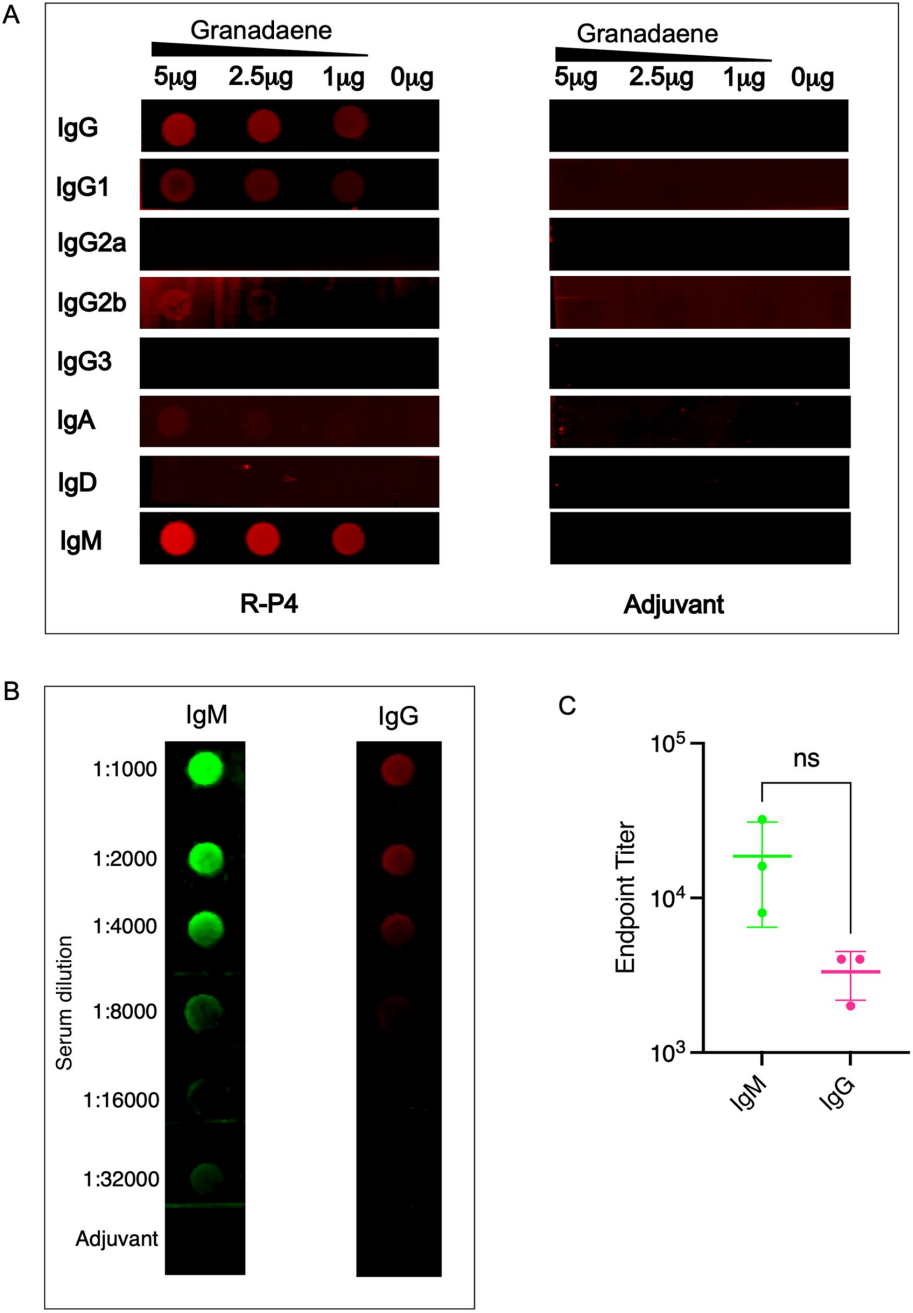
R-P4 vaccination induces IgG and IgM responses in mice. (A) Antibody isotype responses following **R-P4** vaccination. Approx. 5, 2.5 and 1 μg of purified Granadaene was spotted on PVDF membranes and probed with either **R-P4** serum or adjuvant serum (1:62.5 dilution). Membranes were blocked, washed, and probed overnight at RT with either anti-mouse IgG, IgG1, IgG2a, IgG2b, IgG3, IgA, IgD or IgM (1:2500 dilution). Immunoreactive spots were visualized using an infrared imager (LI-COR Biosciences) at 680 nm and images analyzed using Image Studio v5.2.5 software. (B) IgG and IgM endpoint titers induced from **R-P4** vaccination were determined by incubation of Granadaene (1μg, spotted on PVDF membranes as above) with serial dilutions of **R-P4** vaccinated mouse sera, followed by probing with either mouse anti-IgG or IgM antibodies. Endpoint titers were determined as the serum dilution required to exhibit a similar reactivity to Granadaene as that observed with adjuvant control serum. C) Endpoint titers mean ± standard deviation (SD) of IgM and IgG from **R-P4** vaccinated mice (*n = 3*) are shown. ns indicates not significant or p ≥ 0.05, unpaired t test.

We performed immunospot-blot analysis to determine the antibody isotype response to **R-P4** vaccination. To this end, purified granadaene (1–5μg) spotted on PVDF membranes was probed with serum from either **R-P4** -immunized or adjuvant-control mice (*n = 3*/group). The membranes were subsequently probed with infra-red labelled secondary goat anti-mouse antibody to either IgG, or to individual Ig isotypes such as IgG1, IgG2, IgG3, IgA, IgD or IgM. Ig reactive spots were then identified via LI-COR imaging. The results in Fig 3A show that **R-P4** vaccination results in antibody responses to the GBS pigment, Granadaene and predominantly includes IgG (IgG1 and IgG2b) and IgM responses. A very faint signal was also seen towards the IgA secondary antibody. Because **R-P4** or GBS pigment do not bind to commercially available ELISA plates (including hydrophobic or hydrophilic plates), we performed spot blots using serum dilutions of **R-P4** vaccinated mice to determine antibody titers using the secondary IgG and IgM antibodies. Fig 3B shows representative serum dilution blots performed with serum from an adjuvant and a **R-P4** vaccinated mouse. Based on this analysis, titers for IgM and IgG from **R-P4** immunized mice (*n = 3*) are shown in Fig 3C.

### CD4^+^ T cells from R-P4 immunized mice proliferate in a CD1d-dependent manner to R-P4 restimulation

**Fig 4:**
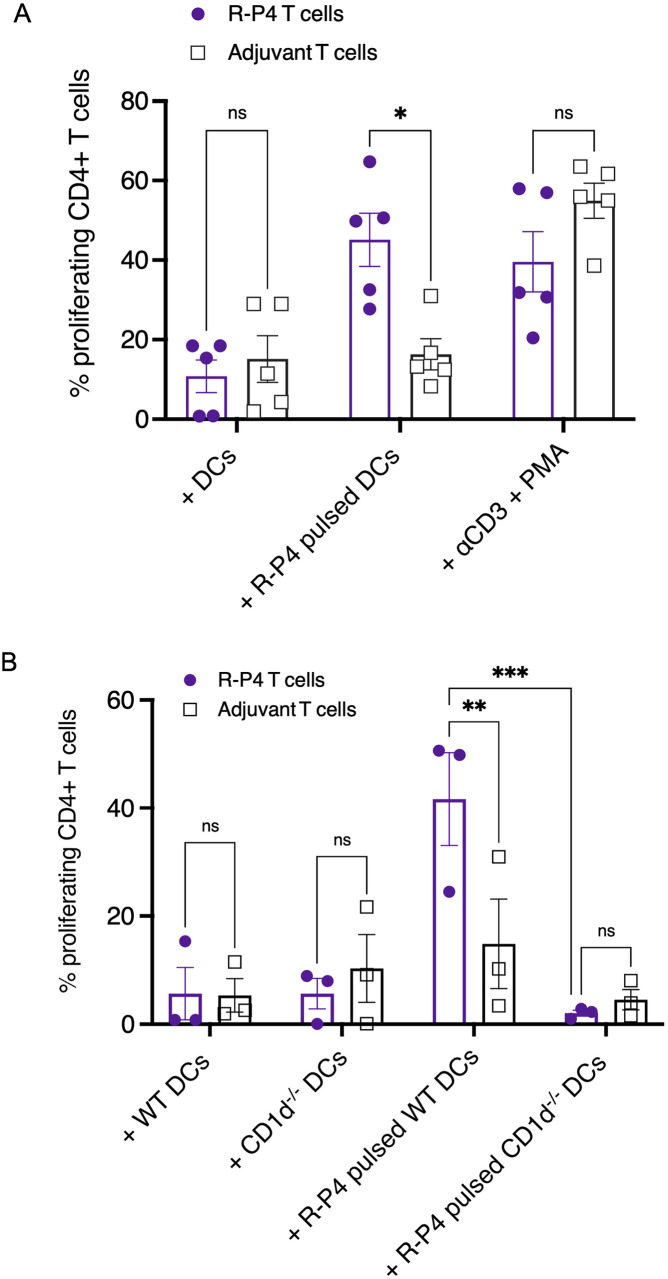
CD4^+^ memory T cells are induced by R-P4 immunization. (A) CD4^+^ T cells isolated from WT mice immunized with **R-P4** or adjuvant only, were stained with CellTraceViolet (CTV) and co-cultured with **R-P4** pulsed WT DCs. As a negative control, T cells were co-cultured with DCs without antigen stimulation. As a positive control for T cell proliferation, T cells were stimulated with anti-CD3 and PMA. After 5–7 days of co-culture, cells were stained for CD4 and assessed for proliferation (as measured by dilution of CTV dye) by flow cytometry. Sidak’s multiple comparison test following 2way ANOVA was used to compare proliferation of T cells between **R-P4** vs adjuvant T cell groups. Data represent mean ± SEM from five independent experiments. * Indicates p < 0.05, ns indicates not significant, or p ≥ 0.05. (B) CD4^+^ T cells isolated from WT mice immunized with **R-P4** or adjuvant only, were stained with CTV and co-cultured with **R-P4** pulsed WT DCs or CD1d^-/-^ DCs. As a negative control, T cells were co-cultured with DCs without antigen stimulation. As a positive control for T cell proliferation, T cells were stimulated with anti-CD3 and PMA. After 5–7 days of co-culture, cells were stained for CD4 and assessed for proliferation (as measured by dilution of CTV dye) by flow cytometry. Data represent mean ± SEM from three independent experiments. Sidak’s multiple comparison test following 2way ANOVA was used to compare T cell proliferation between **R-P4** vs adjuvant groups. ** Indicates p < 0.01, *** indicates p < 0.001, ns indicates not significant, or p ≥ 0.05.

Given that **R-P4** is a lipid and that mice only express the CD1d isotype, we hypothesized that protection conferred by **R-P4** immunization may be partially due to CD1d-restricted T cells. We first determined if **R-P4** immunization induced a memory T cell response by examining if CD4^+^ T cells isolated from **R-P4** immunized mice proliferated in response to **R-P4** stimulation. To this end, CD4^+^ T cells were isolated from splenocytes of **R-P4** immunized or adjuvant treated WT mice and were labeled with CellTraceViolet (CTV) using methods described [66], also see *Material and Methods*). Meanwhile, bone marrow derived dendritic cells (DC’s) were generated from naïve WT mice as described [67] and were pulsed with **R-P4** *in vitro*. Thereafter, CTV-labeled CD4^+^ T cells were co-cultured with **R-P4**-pulsed WT DCs for 5–7 days. CD4^+^ T cell proliferation was then determined by dilution of fluorescent CTV via flow cytometry (See S1 Fig for gating strategy). T cells stimulated with anti-CD3 and phorbol 12-myristate 13-acetate (PMA) acted as a positive control for T-cell proliferation [68,69], while T cells with DCs, but with no antigen stimulation served as the non-proliferation control. The results shown in Fig 4A indicate that upon co-culture with **R-P4**-pulsed DCs, CD4^+^ T cells from **R-P4** immunized mice exhibited significantly increased proliferation when compared to T cells from adjuvant treated mice (see S2 Fig for representative histograms). As expected, no significant differences were observed in the positive controls. Robust proliferation of T cells from **R-P4**-immunized mice compared to adjuvant treated mice indicates that memory CD4^+^ T cells are induced by this antigen which can respond and proliferate to subsequent **R-P4** exposure.

We then tested the role of CD1d in **R-P4** antigen presentation, as lipid antigens can be presented to lipid-specific T cells through CD1d [52–54]. To this end, DCs were generated from bone marrow cells of WT or CD1d knockout mice (CD1d^-/-^) and were pulsed with **R-P4** as above. CD4^+^ T cells were isolated from WT mice that were either immunized with **R-P4** or adjuvant and labeled with CTV, prior to co-culture with **R-P4** stimulated CD1d^-/-^ DCs. Proliferation of **R-P4** specific CD4^+^ T cells was then compared between WT or CD1d^-/-^ DCs. Adjuvant and control groups were included. We observed that CD4^+^ T cells from **R-P4**-immunized mice exhibited decreased proliferation in response to **R-P4** stimulation from CD1d^-/-^ DCs when compared to WT DCs (Fig 4B). Conversely, proliferation of T cells from adjuvant-treated mice was not significantly different following co-culture with **R-P4** pulsed WT or CD1d^-/-^ DCs.

**Fig 5:**
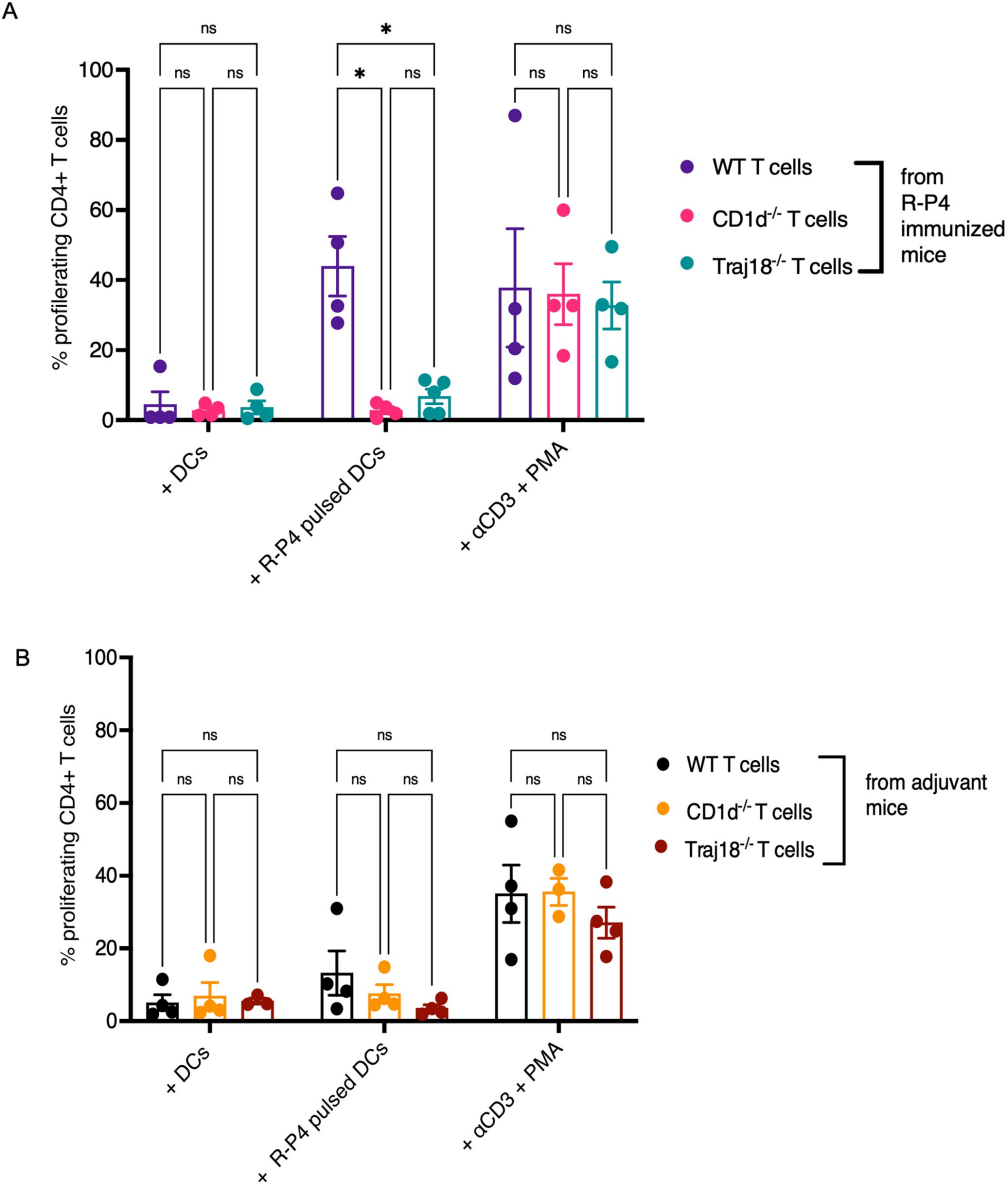
CD4^+^ memory T cells are not induced by R-P4 immunization of CD1d^-/-^ and Traj18^-/-^ mice. CD4^+^ T cells isolated from WT, CD1d^-/-^, and Traj18^-/-^ mice immunized with **R-P4** (A) or adjuvant control (B) were stained with CTV, and co-cultured with **R-P4** pulsed WT DCs. As a positive control for T cell proliferation, T cells were stimulated with anti-CD3 and PMA. As negative controls, treatment groups included unstimulated DCs. After 5–7 days of co-culture, cells were stained for CD4 and assessed for proliferation (dilution of CTV dye) by flow cytometry. Data represent mean ± SEM from at least three independent experiments. Sidak’s multiple comparison test following 2way ANOVA was used to compare proliferation between treatment groups. * Indicates p < 0.05, ns indicates not significant, or p ≥ 0.05.

To evaluate the importance of CD1d-restricted T cells in response to **R-P4** stimulation, we isolated CD4^+^ T cells from **R-P4**-immunized or adjuvant treated mice that included CD1d^-/-^ and Traj18^-/-^ mice. The CD1d^-/-^ and Traj18^-/-^ mice lack CD1d-expressing cells and iNKT cells, respectively. CD4+ T cells were isolated from WT, CD1d^-/-^ or Traj18^-/-^ mice that were immunized with **R-P4** or treated with adjuvant. Proliferation of these cells were examined after co-culture with **R-P4** pulsed WT DCs. The results shown in Fig 5A and 5B indicate that CD4^+^ T cells from **R-P4**-immunized CD1d^-/-^ and Traj18^-/-^ mice had significantly diminished proliferation when compared to CD4^+^ T cells from **R-P4** immunized WT mice. These results indicate that CD1d^-/-^ mice and Traj18^-/-^ mice lack the effector molecule and iNKT cell subsets, respectively that induce **R-P4** vaccine responses. Taken together, our findings suggest that CD1d-restricted iNKT cells maybe important for responses to **R-P4**-immunization.

To determine if iNKT cells were present in the proliferating T cell population after exposure to **R-P4** pulsed DC, we examined their ability to bind to the CD1d tetramer loaded with PBS-57 (an α-GalCer analog). A mock tetramer was included as a control. The results shown in S3 Fig indicate the presence of iNKT cells in the proliferating T cell population in response to **R-P4**.

### CD1d-restricted T cells contribute to protection against GBS systemic infection

**Fig 6:**
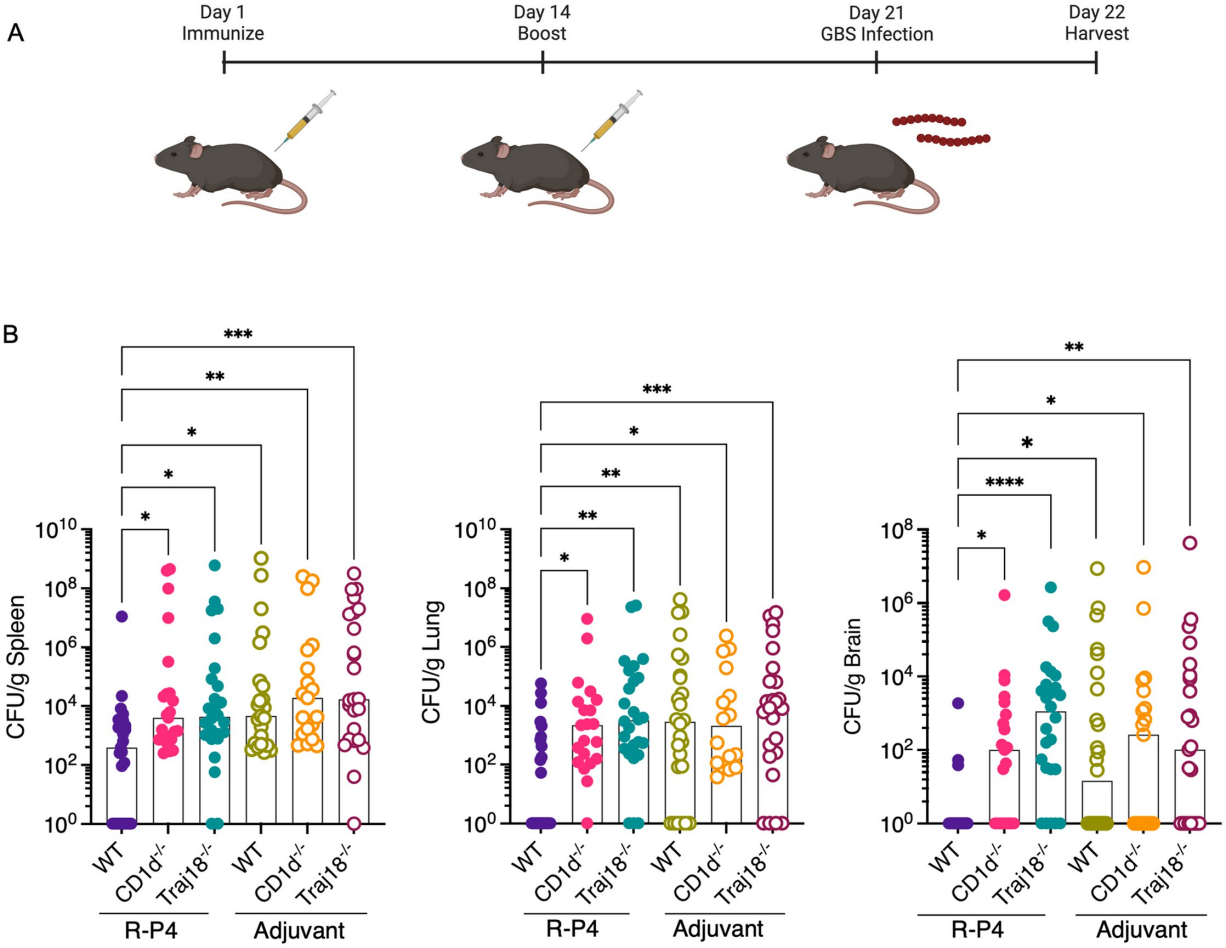
CD1d-restricted iNKT cells contribute to protection against GBS infection *in vivo*. (A) Schematic created using BioRender.com displaying the experimental design. WT, CD1d^-/-^, and Traj18^-/-^ mice were immunized with **R-P4** and boosted 14 days later. At day 21, mice were challenged with HH GBS (WT NCTC10/84). After 24 hours post-infection, mice were euthanized and GBS recovered from spleen, lungs and brain were enumerated using serial dilution and plating on TSA. (B) Bacterial burden in **R-P4** immunized mice. Sample sizes are as follows: *n = 27* WT; *n = 23* CD1d^-/-^; *n = 26* Traj18^-/-^. Adjuvant only control mice were also included. Sample sizes are as follows: *n = 26* WT; *n = 19* CD1d^-/-^;*n = 25* Traj18^-/-^. Data points represent individual mice, with the horizontal line representing the median. Bacterial burden was compared using Kruskal-Wallis test with Dunn’s multiple comparison test. * Indicates p < 0.05, **p < 0.01, *** indicates p < 0.001, **** indicates p < 0.0001.

We next tested if CD1d-restricted T cells are involved in protection against GBS infection *in vivo*. To this end, WT, CD1d^-/-^ and Traj18^-/-^ mice were immunized with **R-P4** or adjuvant as previously described (Fig 6A). The immunized mice were challenged with HH GBS (~1 x 10^8^ CFU, i.p) on day 21 post-initial immunization. At 24 hours after challenge, bacterial burden in the brain, lungs, and spleen was enumerated as described previously. We observed that **R-P4**-immunized CD1d^-/-^ and Traj18^-/-^ mice had elevated CFU in all tissues compared to WT (Fig 6B). Conversely, adjuvant controls had elevated bacterial dissemination across mouse strains (Fig 6B). These data show that the respective lack of CD1d- expression and iNKT cells in CD1d^-/-^ and Traj18^-/-^ mice are associated with increased bacterial dissemination due to the inability to generate protective immunity to HH GBS through **R-P4** immunization. Thus, CD1d+ and iNKT cells contribute to GBS clearance in **R-P4**-immunized mice.

### Adoptive transfer of iNKT cells from R-P4 vaccinated mice diminishes GBS systemic infection

**Fig 7:**
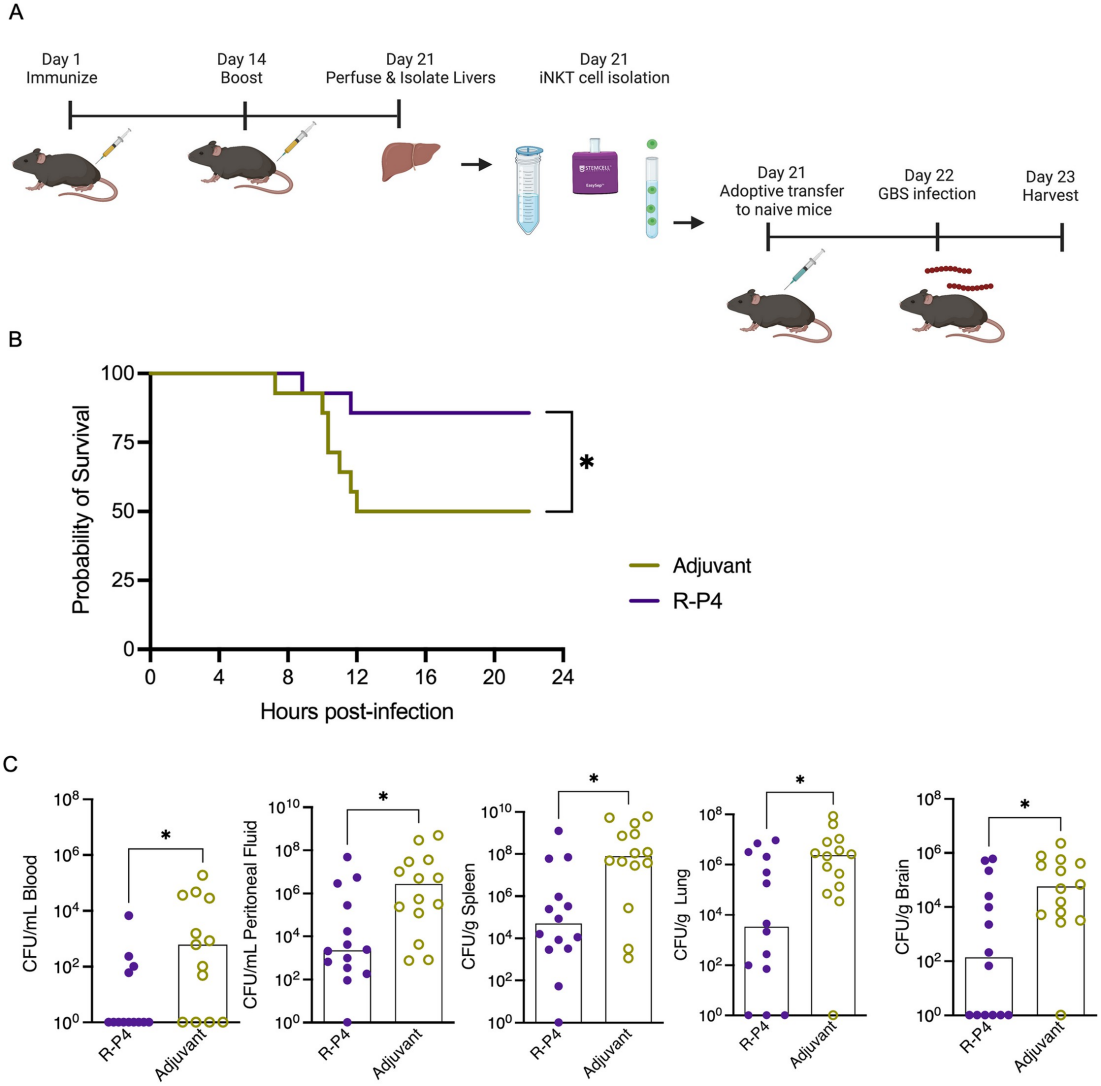
iNKT cells from R-P4 vaccinated mice diminishes GBS infection *in vivo*. (A) Schematic created using BioRender.com displaying the experimental design for the isolation and adoptive transfer of iNKT cells. Mice were immunized i.p with an emulsion of **R-P4** and complete Freund’s adjuvant and boosted 14 days later with an emulsion of **R-P4** and incomplete Freund’s adjuvant. Adjuvant control mice received the adjuvant on the same schedule. On day 21, iNKT cells were isolated from the livers of **R-P4** immunized or adjuvant control mice and the cells were adoptively transferred i.v (retro-orbital route) to naive recipient mice (~1–3 x 10^6^ cells/mouse, *n = 14*/group). One day post transfer, the recipient mice were challenged i.p. with HH GBS (WT NCTC10/84, 1 x 10^7^ CFU, i.p) and mice were monitored for symptoms of morbidity for 24 hrs. (B) Kaplan–Meier survival curve shows that mice that received iNKT cells from R-P4 immunized mice exhibited better survival compared to mice that received iNKT cells from adjuvant-treated mice. * Indicates p < 0.05, Long-rank (Mantel-Cox) test. (C) At 24 hrs post GBS infection or earlier if morbidity was seen, blood, peritoneal fluid, spleen, lungs, and brain were harvested, tissues were homogenized and GBS CFU enumerated using serial dilution and plating. Data points represent individual mice, with the horizontal line representing the median. * Indicates p < 0.05, Mann-Whitney test.

To confirm the importance of iNKT cells in **R-P4** mediated protection against GBS infection, we performed adoptive transfer of these cells to naïve mice followed by GBS infection (see Fig 7A for scheme). Previous studies have noted that livers have greater number of iNKT cells when compared to the spleen [70] and we observed similar trends (30% vs 5%, in livers vs spleen, respectively). Thus, we isolated iNKT cells from livers of **R-P4** vaccinated mice or mice that received the adjuvant alone. Flow cytometry analysis confirmed that 99% of the isolated cells were TCRbeta+ and were enriched for iNKT cells, as shown by 30–50% of the cells recognizing the α-GalCer tetramer (S4 Fig). The enriched iNKT cells were administered i.v (via the retro-orbital route) to non-immunized recipient mice at a dose of 1–3 x 10^6^ cells per mouse. Controls included naïve recipient mice that received iNKT cells from adjuvant immunized mice. Approximately 24 hours after adoptive transfer of these cells, mice were challenged with HH GBS (1 x 10^7^ CFU, i.p). The recipient mice were monitored for signs of morbidity and were euthanized when morbid or at 24 hrs post infection. Peritoneal fluid, blood, lungs, spleen, and brain were collected from the infected mice, and tissues were homogenized and GBS CFU was enumerated. The results shown in Fig 7B and 7C indicate that mice that received iNKT cells from **R-P4** immunized mice exhibited better survival and significantly reduced GBS burden in the blood, peritoneal fluid, spleen, lungs, and brain, compared to control mice that received iNKT cells from adjuvant controls. Collectively, these data suggest that iNKT cells that have been exposed to **R-P4** immunization are important for protection against GBS infection.

### iNKT cells from R-P4 vaccinated mice exhibit increased cytokine responses

**Fig 8:**
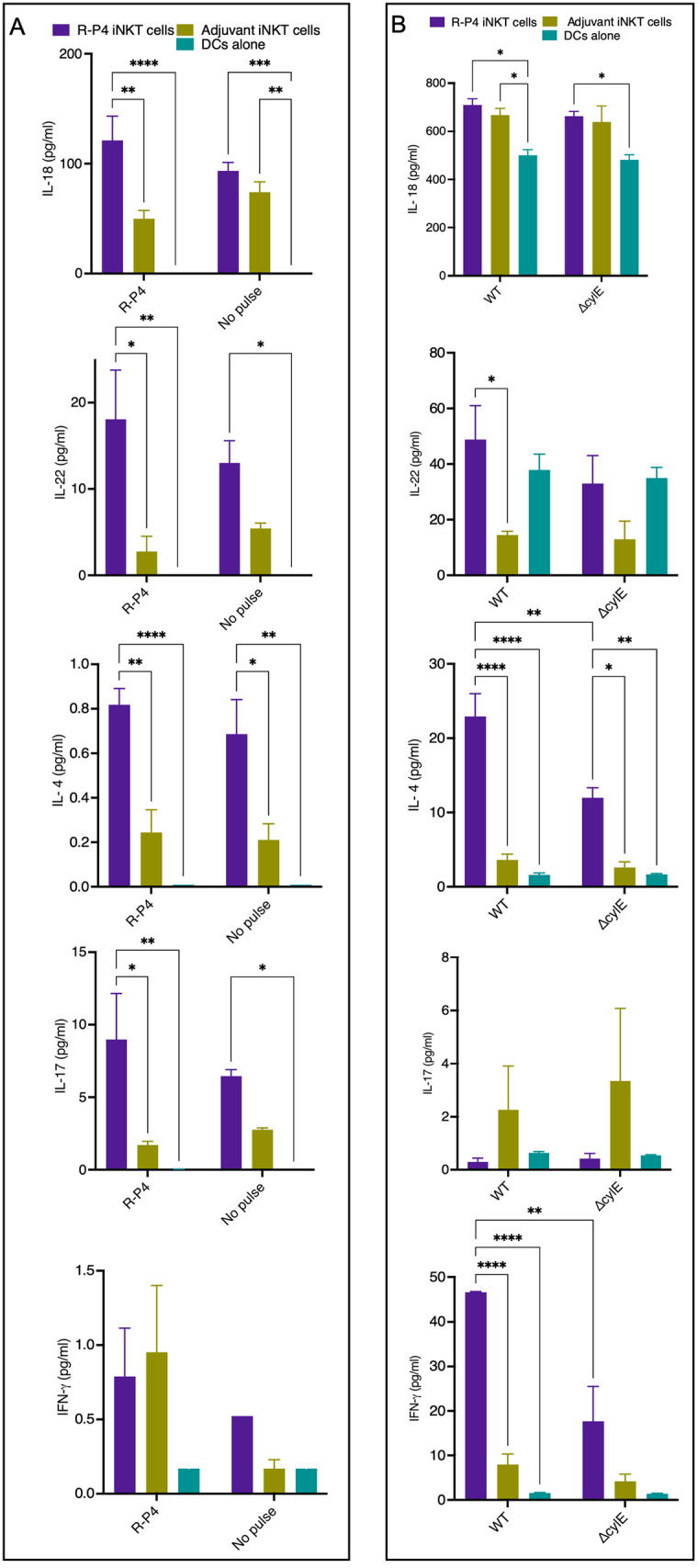
iNKT cells exhibit cytokine responses to R-P4 antigen and GBS stimulation. (A) iNKT cells isolated from WT mice immunized with **R-P4** or adjuvant were co-cultured with **R-P4** pulsed WT DCs. As controls, T cells were co-cultured with DCs without antigen stimulation or DCs were cultured alone. After 5 days of co-culture, cytokine concentrations in supernatants were determined using multiplex assays. Data represent mean ± SEM. Tukey’s multiple comparison test following 2-way ANOVA. * Indicates p<0.05. (B) iNKT cells isolated from WT mice immunized with **R-P4** or adjuvant were co-cultured with WT DCs pulsed with UV-killed WT GBS (NCTC10/84). As controls, T cells were co-cultured with DCs pulsed with UV-killed isogenic pigment/hemolysin deficient GBS (Δ*cylE*) or DCs were cultured alone. After 5 days of co-culture, cytokine concentrations in supernatants were determined using multiplex assays. Data represent mean ± SEM. Tukey’s multiple comparison test following 2way ANOVA. * Indicates p<0.05, ** indicates p < 0.01, *** indicates p<0.001, *** indicates p<0.0001, ns indicates not significant, or p ≥ 0.05.

We also assessed the cytokines responses of iNKT cells in **R-P4** vaccinated mice. Therefore, iNKT cells isolated from **R-P4** vaccinated mice or adjuvant control mice were co-cultured with bone marrow-derived dendritic cells (DCs) that were pulsed with either **R-P4** or buffer (*i*.*e*., no pulse). After 5 days of co-culture, supernatants were harvested, and cytokine concentrations were determined using Luminex assays. The results shown in Fig 8A indicate that in response to **R-P4** antigen, iNKT cells produced significantly more IL-18, IL-22, IL-4, and IL-17 compared to controls. These experiments were then repeated using UV killed HH GBS (WT NCTC10/84) or isogenic hemolytic pigment deficient GBS (Δ*cylE*) and the results are shown in Fig 8B. Interestingly, the iNKT cell response to HH GBS also included a significant increase in IL-18, IL-22, IL-4 and even IFN-γ compared to nonpigmented GBSΔ*cylE*. We predict that slight differences in cytokines responses by iNKT cells upon restimulation with **R-P4** versus HH GBS may in part be due to differences in antigen presentation by the DCs. Taken together with previous observations that IL-4 induces IgG1 class switching [71] and that IL-4 and IFN-γ are important for both non-cognate and cognate iNKT cell help to antigen specific B cells [72], these findings indicate that **R-P4** vaccination promotes iNKT cell responses to limit GBS dissemination.

### Increased inhibition of pigment hemolysis from R-P4 vaccinated WT mice

**Fig 9:**
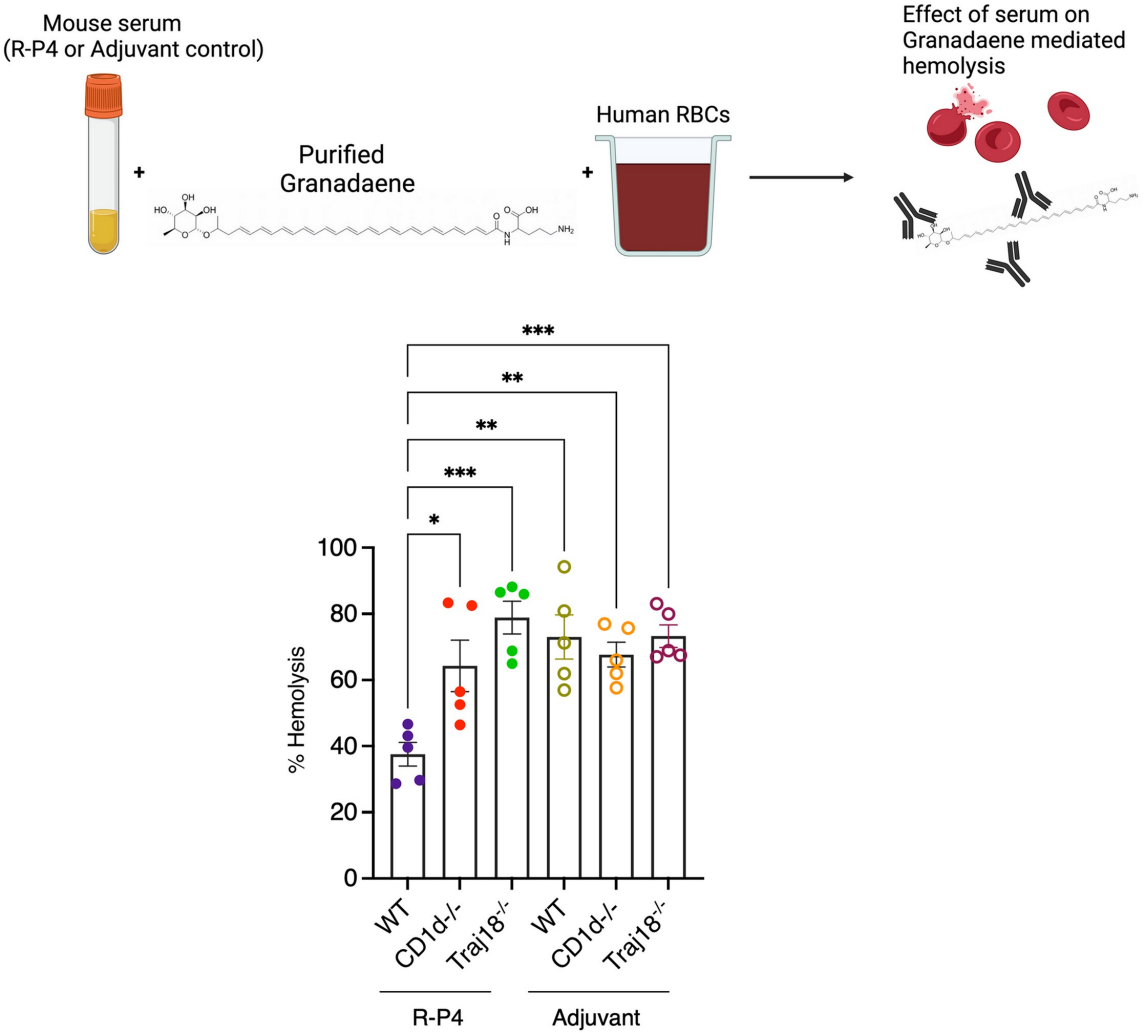
Decreased hemolysis inhibition by R-P4-immunized CD1d^-/-^ and Traj18^-/-^ mice. Schematic created using BioRender.com displaying the experimental design wherein diluted serum from **R-P4** immunized or adjuvant control WT, CD1d^-/-^ and Traj18^-/-^ mice was incubated with Granadaene (1.25mM) prior to hemolysis assays with human red blood cells. Hemoglobin release in cell supernatants was measured (absorbance at 420 nm), and percent hemolysis relative to Triton X-100 (0.1%) control (100% hemolysis) and PBS-treated negative controls (0% hemolysis) was calculated. Data represent mean ± SEM from five samples/group. Tukey’s multiple comparison test following ANOVA. * Indicates p < 0.05, ** indicates p < 0.01, *** indicates p < 0.001.

To determine if CD1d^-/-^ and Traj18^-/-^ affected antibody production that inhibits pigment mediated hemolysis, mice were vaccinated with **R-P4** or adjuvant as described earlier. Serum from **R-P4** or adjuvant immunized WT, CD1d^-/-^ and Traj18^-/-^ mice were exposed to GBS pigment (1.25mM) respectively, prior to performing hemolysis assays as described [20]. The results shown in Fig 9 indicate that serum from **R-P4**-immunized WT mice exhibited greater inhibition of pigment mediated hemolysis when compared to **R-P4** immunized CD1d^-/-^ or Traj18^-/-^ mice. As expected, adjuvant control mice did not inhibit pigment hemolysis. These data further confirm the importance of CD1d^+^ and iNKT cells in **R-P4** mediated protection against GBS pigment.

### R-P4 immunization provides protection against ascending GBS infection

**Fig 10:**
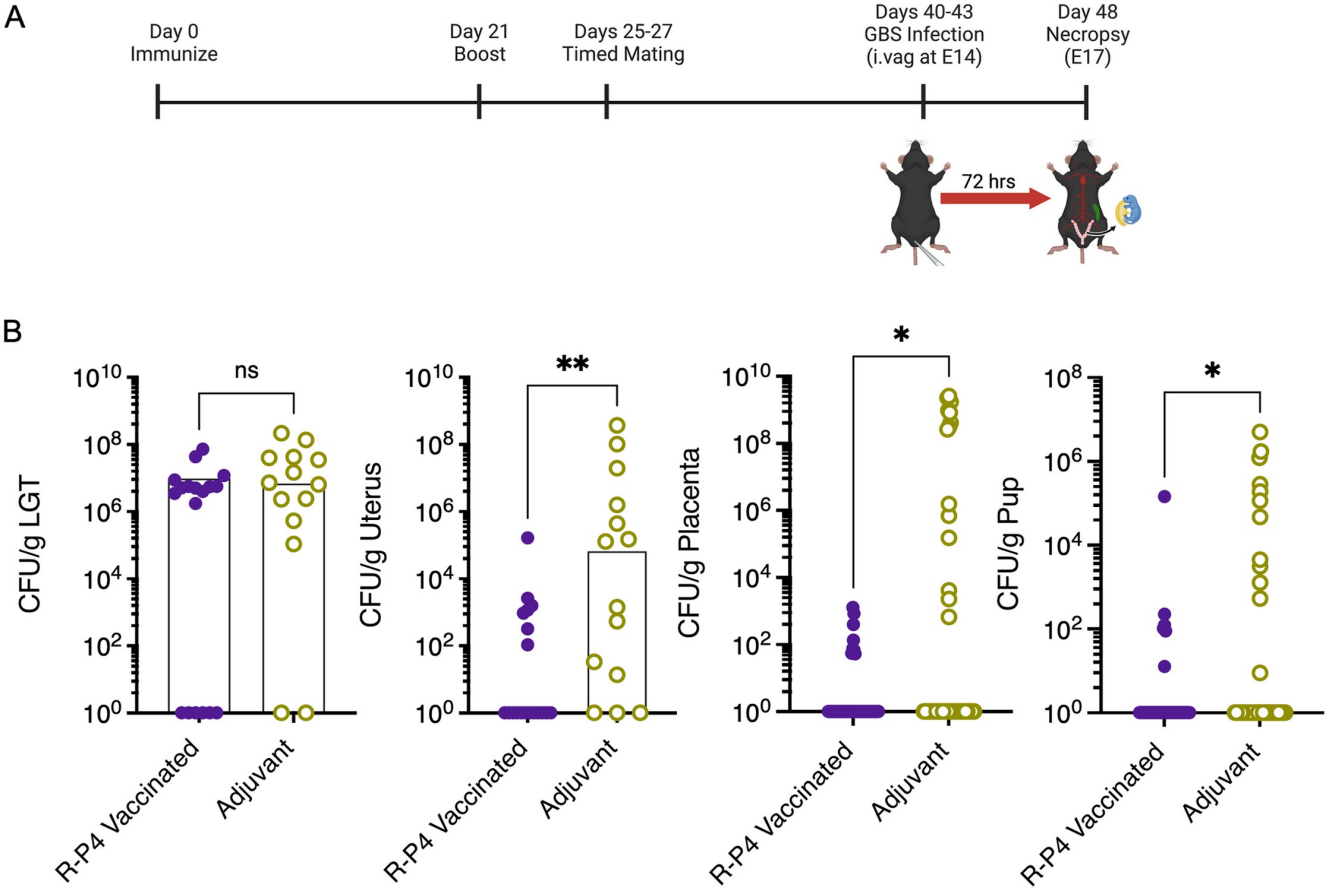
Maternal R-P4 immunization diminished GBS ascending infection. (A) Schematic created using BioRender.com displaying the timeline for pregnancy-associated vaginal GBS challenge following **R-P4** immunization. After their final vaccine boost, mice were mated and monitored for pregnancy. On day 14 of pregnancy, mice were intravaginally challenged with 10^8^ CFU of GBS (WT NCTC10/84). (B) Dams were euthanized at ~72 hours post-infection and blood, lower genital tract (LGT), uterus, proximal and distal placentas and their pups were collected, homogenized, and plated for CFU enumeration. Medians are indicated with circles representing values from individual mice. Statistical differences were determined by Mann-Whitney test (sample sizes: *n = 14* adjuvant, *n = 18* adjuvant + **R-P4**). * Indicates p < 0.05, ** indicates p < 0.01, ns indicates not significant, or p ≥ 0.05.

We then examined whether **R-P4** immunization can diminish ascending GBS infection in pregnant mice. Female WT mice were immunized with **R-P4** or adjuvant as previously described (see Fig 10A for scheme). The immunized and control mice were then mated for pregnancy. At day 15 (E15) of pregnancy, mice were vaginally inoculated with approximately 10^8^ CFU of HH GBS using methods described [73,74]. The mice were then monitored for signs of preterm birth (defined as vaginal bleeding or pups in canal or cage) for up to 72 h post GBS inoculation. Upon signs of preterm birth or at 72 h post-inoculation (whichever occurred first), the mice were euthanized, a midline laparotomy was performed, and tissues (lower genital tract, uterus, placenta, pups) were excised to estimate bacterial burden. The results shown in Fig 10B indicate that **R-P4** immunized WT mice had reduced rates of ascending GBS infection in uterine and placental tissues compared to adjuvant controls. Additionally, **R-P4** immunization decreased GBS dissemination to fetal pups. Together, these data indicate that **R-P4** immunization diminishes ascending GBS infection in a pregnant murine model.

**Fig 11:**
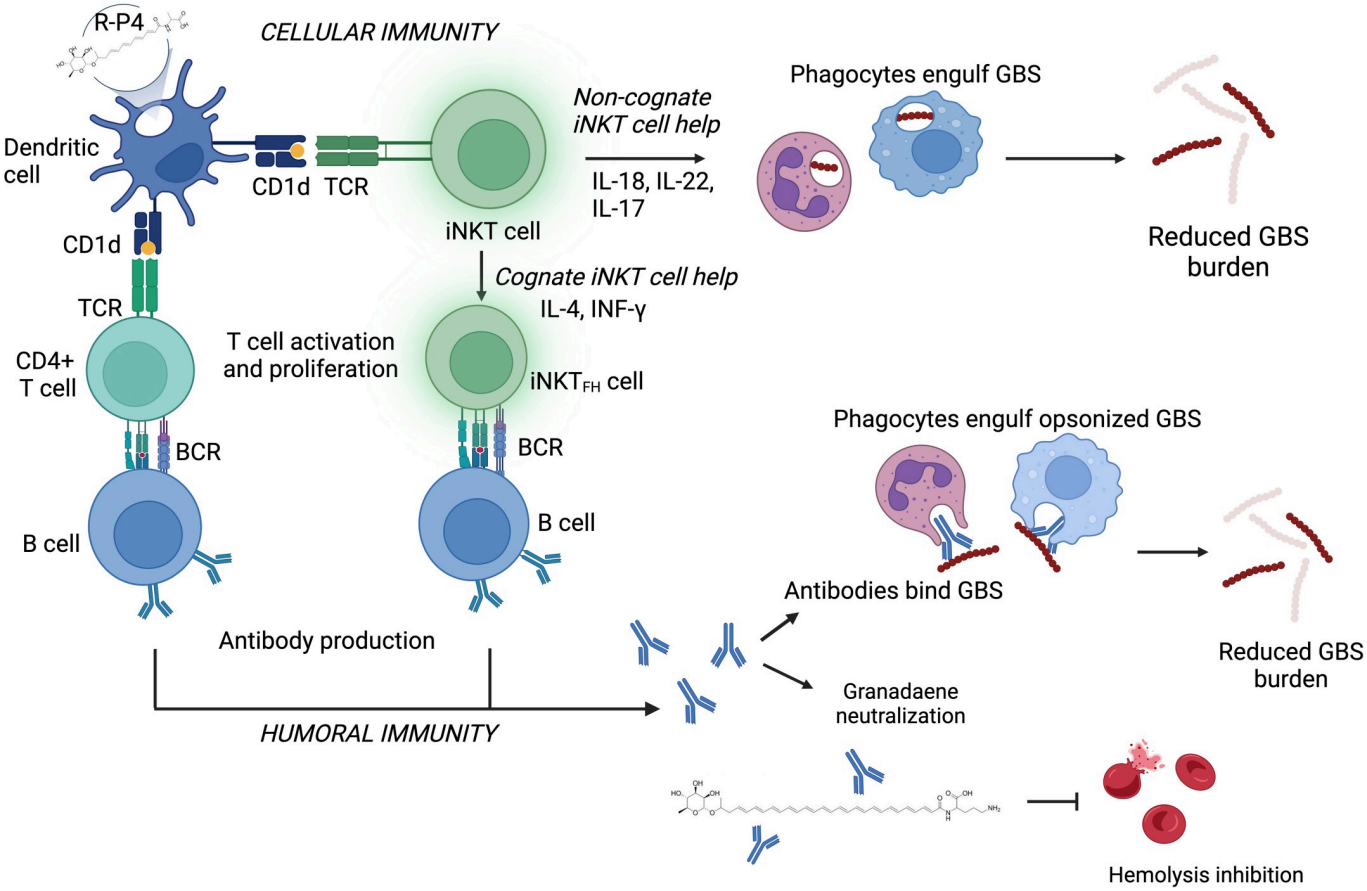
Model demonstrating the importance of antibody and T cell responses in R-P4 mediated immune protection against GBS infection. Model created using BioRender.com showing humoral and cellular immunity in **R-P4**-mediated protection against GBS infection. **R-P4** presentation by dendritic cells requires CD1d, which promotes T cell proliferation resulting in antibody production (IgG and IgM) via B cells. CD1d restricted T cells such as iNKT cells also are important for **R-P4** mediated protection, which can provide noncognate help through production of inflammatory cytokines such as IL-18, IL-22 and IL-17 resulting in recruitment of phagocytes that dimmish GBS burden. Alternatively, iNKT cells can provide cognate help via IL-4 and INF-γ promoting direct interaction between iNKT follicular helper cells (iNKT_FH_) with T cells resulting in antibody production that dimmish cytotoxicity and GBS dissemination.

In summary, our results indicate that a non-toxic lipid analog such as **R-P4** elicits protective immunity against the GBS pigment, granadaene through antibody production and T cell responses that are dependent on CD1d and iNKT cells (see model in Fig 11).

## Discussion

Little is known about the adaptive immune response to lipid antigens including the GBS hemolysin, granadaene. To date, GBS hemolysin-specific antibodies or T cell responses have not been reported in patient samples, which may be explained by the cytolytic effects of hemolysin on B cells and T cells [20]. This cytotoxic activity against host immune cells [18–21,75] poses significant challenges to the development of a vaccine that targets granadaene. Despite these obstacles, the synthesis and identification of **R-P4** as a non-cytotoxic analog of granadaene enabled its use in a pre-clinical vaccine model [20]. Here, we show that immunization with **R-P4** induces protective antibody and T cell responses. Antibody responses generated from **R-P4** immunization facilitate GBS clearance through opsonophagocytic killing by phagocytes such as neutrophils and macrophages. These antibodies contribute to protection *in vivo* and is further supported by our observations in naïve mice wherein mice that received **R-P4** immune serum exhibited reduced bacterial burden compared to mice that received adjuvant control serum. However, it is also likely that **R-P4** immune serum may diminish GBS pigment associated host cytotoxicity *in vivo*, which could further restrict bacterial dissemination.

We demonstrate that in response to **R-P4** stimulation, CD4^+^ T cell proliferation is dependent on CD1d expression by antigen presenting cells such as DCs and the proliferating T cell population included iNKT cells. In the absence of the CD1d or iNKT cells, protection from GBS systemic infection following **R-P4** immunization is lost. CD1d is thought to mediate direct or indirect recognition of foreign lipid antigens by iNKT cells through the following proposed mechanisms [76]. iNKT cells can recognize foreign antigen presented by CD1d through direct TCR engagement, resulting in iNKT cell activation [58,77,78]. Alternatively, foreign antigens can be indirectly recognized through cytokine-dominated iNKT cell activation. In this case, Toll like receptor (TLR) activation of antigen presenting cells (APCs) results in the production of proinflammatory cytokines. In conjunction with TLR-induced inflammatory cytokines, TCR engagement of either a microbial lipid antigen or self-lipid antigen is required for iNKT cell activation [79–81]. We speculate that both cognate and non-cognate iNKT cell interactions are involved in protection following R-P4 immunization. Although iNKT cells were not the only T cells responding to **R-P4**, *in vivo* data from Traj-18 knockout mice indicate that these cells are important for protection. Furthermore, adoptive transfer of iNKT cells from **R-P4** vaccinated mice significantly diminished GBS infection compared to controls. Luminex assays from the *in vitro* studies revealed that iNKT cells exhibit Th17 (IL-17, IL-22), Th-2 (IL-4) and Th1 (IFN-γ, IL-18) response to **R-P4** and HH GBS, as observed previously with other lipids [82,83].

Immunospot blots revealed that **R-P4** vaccination results in IgG (IgG1, IgG2B) and IgM response to GBS pigment. As **R-P4** or pigment do not bind to commercially available ELISA plates (including hydrophobic or hydrophilic plates) we were thus unable to quantify antibody levels using traditional ELISA methodologies using these antigens. However, using immune spot blots analysis, we determined that antibody titers ranged from 2000–8000 for IgG and 8000–32000 for IgM, respectively. It is likely that the IgM response also aids in limit GBS dissemination in the systemic model and perhaps even in the pregnancy model as vaccinated mice that had no GBS CFU in uterine tissues also had no GBS in fetal tissues. Future studies with improved granadaene analogs and using higher order animal models of pregnancy such as nonhuman primates will provide additional insight into mechanisms important for prevention of GBS infections during pregnancy.

Lipid-based vaccines are promising interventions as they circumvent certain limitations that arise from vaccine responses to peptide antigens. Peptide antigens are presented on MHC/HLA and due to polymorphisms in MHC/HLA genes [84], the effectiveness of peptide-specific vaccine responses may vary depending on the MHC haplotype of the individual [85,86]. In contrast, CD1 molecules are non-polymorphic [87], thus minimizing differences in CD1-restricted T cell responses amongst individuals [88]. While our study underscores the importance of CD1d in the murine response to **R-P4** immunization, mice only express CD1d. Therefore, investigation into CD1-restricted T cell responses in humans would provide additional insight on **R-P4** mediated protective immune mechanisms, and would delineate the involvement of the 4 human CD1 isotypes (CD1a, CD1b, CD1c, CD1d), which are all involved in the surface presentation of lipid antigens [52–54].

Our studies show that **R-P4** immunization decreased systemic and pregnancy-associated GBS infections. This is noteworthy as majority of GBS cases occur in pregnant women and their neonates. [89,90]. Thus, a vaccine that curtails GBS infection during pregnancy is of high priority [91]. Despite the recent abundance of pre-clinical GBS vaccines targeting either the capsular polysaccharide (CPS), surface proteins, or in this case lipid antigen, protective capacity during pregnancy varies greatly and multiple challenges remain. First, generating cross-protection against the major GBS serotypes can be difficult, particularly for CPS-based strategies which must be multivalent to achieve optimal protection [92,93]. Pre-clinical protein-based formulations simplify this issue by targeting surface antigens that are more highly conserved across GBS strains; however, achieving strong protection depends on the adjuvant used and the vaccination route [94–98]. Although our **R-P4** vaccine utilized a strong adjuvant that is not approved for use in humans, the vaccine elicited the production of protective and functional antibodies that diminished systemic and ascending GBS infection. **R-P4** immunized dams exhibited significantly reduced rates of ascending infection, as GBS was detected from uterine samples in only 38% of **R-P4** immunized mice, versus 78% of adjuvant controls. While **R-P4** vaccination diminished GBS infection in the systemic and pregnancy models of GBS infection, complete elimination of bacteria was not observed. This may be due to the route of vaccination, severity of the disease models or limitations in vaccine efficacy. To gain fuller insight to the protective capacity of **R-P4**, future studies should assess **R-P4** with adjuvants approved for use in humans and determine if maternal vaccination induces transfer of antibodies to the neonate to prevent neonatal disease. Additional synthetic non-toxic analogs of granadaene may also prove useful in vaccination strategies.

In conclusion, our findings define protective antibody mechanisms driven by **R-P4** immunization, which decreased the severity of GBS infections. In addition, we show that **R-P4** immunization induces a memory T cell response which is CD1d-dependent and that iNKT cells are important for protection against GBS infection. Collectively, these findings provide a better understanding of the mechanisms that confer protection through **R-P4** immunization and will inform the design of therapeutic strategies that target GBS hemolysin and other bacterial lipid cytotoxins.

## Materials and methods

### Ethic statement

Formal written informed consent for donations of human blood was obtained from patients, per the Principles in the WMA Declaration of Helsinki and Dept. of Health and Human Services Belmont Report. The study was approved by the Seattle Children’s Research Institute Institutional Review Board (protocol #11117). Children under the age of 18 were not recruited for donation of human blood and did not participate in the study.

All animal experiments were approved by the Seattle Children’s Research Institutional Animal Care and Use Committee (protocol# 00036) and performed in strict accordance with the recommendations in the *Guide for the Care and Use of Laboratory Animals*, *8*^*th*^ *ed*.

### Bacterial strains

Unless otherwise noted, the WT GBS strain NCTC10/84 used in this study is a serotype V (sequence type 26 clone) clinical isolate obtained from an infected newborn [42]. GBS hemolysin was isolated from the WT GBS strain A909 and isogenic Δ*cylE* mutant was included as a control. Overnight GBS cultures were grown in tryptic soy broth (TSB) at 30°C in 5% CO_2_ and then sub-cultured 1:10 in TSB. Subcultures were grown to optical density at 600nm (OD_600_) of 0.3. Bacterial strains were washed twice with PBS and resuspended to 1 x 10^9^ CFU/mL (unless otherwise mentioned) in PBS before used in experiments.

### Isolation of Granadaene

Granadaene was isolated from WT A909 and isogenic Δ*cylE* was included as a control as described [20,41]. Briefly, A909 WT and Δ*cylE* were grown in 2L of Granada media at 37°C for 24 hours. Bacterial pellets were washed with HPLC-grade water three times and then with DMSO twice. Granadaene was extracted from the bacterial pellets using DMSO + 0.1% trifluoroacetic acid (TFA). The crude extracts were column-purified using high pressure liquid chromatography (HPLC) using a Vydac 214TP C4 column. Pigmented fractions were pooled and granadaene was precipitated from the purified factions with NH_4_OH, lyophilized until dry and stored at -80°C. For hemolysis inhibition assays, lyophilized granadaene was dissolved in DMSO + 0.1% TFA + 20% starch (DTS) and diluted in PBS to indicated concentration, as previously described [20,41].

### Synthesis of R-P4

**R-P4** was synthesized as previously described [20] and the entire procedure for the synthesis of **R-P4** is provided in S1 Text. ^1^H NMR spectrum of purified samples was used to confirm identity and purity of **R-P4** (S5 Fig).

### Mouse strains

Six- to 8-weeks old WT C57BL/6J, isogenic CD1d knockout (CD1d^-/-^) and Traj18 knockout (Traj18^-/-^) mice were obtained from Jackson Laboratories. Both male and female mice were used in all experiments unless noted otherwise. All mice were maintained under specific pathogen-free conditions. Genotypes of mice bred in-house were confirmed by PCR before use.

### R-P4 immunization and systemic GBS inoculation

Six- to 8-weeks old mice (WT, CD1d^-/-^ or Traj18^-/-^) obtained from Jackson Laboratories were used for immunization studies as described previously [20]. Approximately equal number of male and female mice were represented in each immunization group. First, immunizations with **R-P4** emulsions were prepared by mixing **R-P4** (20mM dissolved in PBS) and complete Freund’s adjuvant (CFA, Invivogen) at a 1:1 ratio. Emulsions (100μL) were administered to mice intraperitoneally (i.p). Two weeks after the initial immunization, booster doses (100μL) were prepared by mixing **R-P4** (20mM dissolved in PBS) and incomplete Freund’s adjuvant (IFA, Invivogen) at a 1:1 ratio and administered to mice i.p Adjuvant-only emulsions were prepared by mixing sterile PBS with the appropriate adjuvant at a 1:1 ratio and these were administered to mice i.p according to the same schedule as **R-P4**-immunizations. One week after the final immunization, mice were challenged (i.p) with approximately 1 x 10^8^ CFU of NCTC10/84 GBS in 100uL of sterile PBS. At 24 hours post-GBS infection, mice were euthanized and brains, lungs, and spleens were collected in 1mL of sterile PBS and homogenized. GBS CFU in each tissue was enumerated by serial dilution and plating on tryptic soy agar (TSA, Difco Laboratories).

### Serum transfer to non-immunized mice

One week after the final immunization of **R-P4**, blood was collected from **R-P4** immunized and adjuvant treated WT mice. Blood collected in serum separator tubes (BD) was allowed to coagulate at room temperature for 30 minutes and then centrifuged at 10,000 x g for 90 seconds. Serum was collected from above the separator gel. Immune serum was vortexed and immediately administered intravenously (i.v, via the tail vein) to non-immunized, six- to 8-weeks old WT mice. One day later, mice were challenged (i.p) with approximately 1 x 10^7^ CFU of NCTC10/84 GBS in 100uL of sterile PBS. Approximately 24 hours post-infection, mice were euthanized, and brains, lungs, and spleens were collected in 1mL of sterile PBS and homogenized. GBS CFU in each tissue was enumerated by serial dilution and plating on TSA.

### Isolation of neutrophils from human blood

Blood from 3 healthy human donors was collected into EDTA tubes (BD Bioscience) and pooled. Immediately following collection, neutrophils were isolated from the blood using Human Whole Blood Neutrophil Isolation Kit (Miltenyi) following manufacturer’s instruction. Cells were then pelleted, and any residual RBCs were removed by resuspending the cell pellet in 15mL of 1x RBC lysis solution (eBiosience) for 15 minutes at room temperature. The RBC lysis solution was quenched and washed with RPMI 1640 medium (Corning) containing L-glutamine, and viable neutrophils were counted following staining by Trypan blue (Gibco).

### Opsonophagocytosis and killing assay

Opsonophagocytosis and killing assay with human neutrophils was performed using methods described with modifications [99]. Immune serum obtained from **R-P4**-immunized, or adjuvant control mice was heat-inactivated by incubation at 56°C for 30 minutes. GBS WT NCTC10/84 was grown to mid-exponential growth phase (OD_600_ = 0.3) and washed twice in sterile PBS. Then, 1 x 10^4^ CFU of GBS in 50μL DMEM was pre-treated with inactivated **R-P4**-immunized or adjuvant control immune serum (diluted 1:30) for 30 minutes at 37°C with shaking. These samples were then incubated with 1 x10^6^ neutrophils and 10% baby rabbit complement (Cedarlane) for 60 minutes at 37°C. Pre-immune serum was also included as a negative control. GBS CFU recovered before and after 90 minutes of incubation was enumerated using serial dilution and plating on TSA. Percent opsonophagocytic killing of GBS was calculated by [(CFU/mL_t = 0_ –CFU/mL_t = 90_) / CFU/mL_t = 0_] x 100%.

Opsonophagocytosis and killing assay with mouse macrophages was performed using methods described [65] with minor modifications. Briefly, GBS WT NCTC10/84 cultured in TSB was washed in Hanks Balanced Salt Solution and adjusted to a final concentration of 1x10^5^ CFU/50μl (t = 0). Bacteria were opsonized by incubation with inactivated serum from **R-P4** vaccinated mice (final dilution 1:30) in the presence of 10% baby rabbit complement in a total volume of 100μl for 60 min at 4°C. Controls included adjuvant and pre-immune sera. After opsonization, 100μl of RAW264.7 murine macrophage cells were added (2 x 10^6^ cells/100μl) and incubated in a rotating shaker at 37°C for 60 min (t = 120). After final incubation, 1% Triton-X was added to lyse cells. GBS CFU recovered before and after 120 minutes of incubation was enumerated using serial dilution and plating on TSA. Percent opsonophagocytic killing of GBS was calculated by [(CFU/mL_t = 0_ –CFU/m _t = 120_) / CFU/mL _t = 0_] x 100%.

### Immunoglobulin isotype analysis

Analysis of antibody isotypes raised following **R-P4** vaccination was performed as described previously [20] with the following modifications. Granadaene in DTS was diluted in PBS and approx. 1, 2.5 and 5μg was spotted to an Immobilon-P PVDF membrane (EMD Millipore Corp) using a Bio-Dot suction manifold (Bio-Rad Laboratories). Membranes were blocked with Intercept PBS Blocking buffer (LI-COR Biosciences) for 1 hr at room temperature and subsequently incubated with either **R-P4** or adjuvant mouse serum (diluted 1:62.5 in Intercept Antibody Diluent (LI-COR Biosciences) overnight at room temperature. Membranes were then washed in PBST (PBS +0.02% Tween-20) and probed overnight at room temperature with either IRDye 680 anti-mouse IgG, IRDye 680 anti-mouse IgG1, IRDye 680 anti-mouse IgG2a, IRDye 680 anti-mouse IgG2b, IRDye 680 anti-mouse IgM (LI-COR Biosciences), anti-mouse IgG3-Dylight 680 (Novus Bio), anti-mouse IgA-BIOT (Southern Biotech) or anti-mouse IgD-BIOT (Invitrogen). All anti-mouse secondary antibodies were diluted 1:2500 in PBST prior to incubation. Membranes probed with anti-mouse IgA-BIOT or anti-mouse IgD-BIOT were washed in PBST and incubated for an additional overnight step at room temperature with IRDye 680-Streptavidin (LI-COR Biosciences). Immunoreactive spots were visualized using an infrared imager (LI-COR Biosciences) at 680 nm and images analyzed using Image Studio v5.2.5 software.

### Endpoint titer determination

PVDF membranes containing 1μg of granadaene were blocked in Intercept PBS Blocking buffer (LI-COR Biosciences) as described above. Membranes were subsequently incubated with serial dilutions of **R-P4** serum ranging from 1:1000–1:32000 and probed by simultaneous incubation with IRDye 680 anti-mouse IgG and IRDye 800 anti-mouse IgM (LI-COR Biosciences). Total IgG and IgM endpoint titers were considered as the serum dilution required to show an identical reactivity to granadaene as seen with mouse adjuvant control sera (1:250 dilution).

### T cell proliferation assay

Bone marrow derived dendritic cells were generated from WT and CD1d^-/-^ mice as described [67]. Briefly, cells were cultured in RPMI-G (supplemented with 10% FBS, 100 IU/mL penicillin, 100μg/mL streptomycin, and 20 ng/mL recombinant murine GM-CSF) at 6 x 10^5^ cells/ml in non-TC-treated petri dishes. Cultures were maintained for 10 days, with feeding on days 3 and 7 and complete replenishment of media on day 7. On day 10, supernatants containing DCs were seeded at 5 x 10^4^ cells/well in RPMI-G (supplemented with 10% FBS, 100 IU/mL penicillin, 100μg/mL streptomycin, 10ng/mL recombinant GM-CSF). The following day, the DCs were pulsed with **R-P4** (10μM) in serum-free RPMI overnight. Pulsed DCs were washed twice with RPMI prior to T cell co-culture.

CD4^+^ T cells were isolated from splenocytes of WT, CD1d^-/-^, Traj18^-/-^ mice immunized with **R-P4** or adjuvant control, using the EasySep CD4^+^ T cell isolation kit (StemCell Technologies), per manufacturer’s instructions. CD4^+^ T cells (1 x 10^6^ cells/mL) were labeled with 5μM CellTraceViolet (CTV) per for 30 minutes at 37°C and quenched with RPMI with 10% FBS. The labeled CD4^+^ T cells were resuspended in RPMI-G (supplemented with 10% FBS, 100 IU/mL penicillin, 100μg/mL streptomycin), and were added to each well of DCs at a ratio of 1:10 (5 x 10^4^ DC:5 x 10^5^ T cells). Negative controls included T cells alone (no co-culture) and T cells with unpulsed DCs (no antigen stimulation). As a positive control for T cell proliferation, T cells from **R-P4** immunized or adjuvant mice were stimulated with anti-CD3 (0.5μg) and PMA (10ng/mL) [68,69]. Proliferation was assessed as dilution of CTV after 5–7 days of co-culture by flow cytometry. Briefly, cells were washed with FACS buffer (1mM EDTA, 25 mM HEPES, 0.1% BSA in PBS) and incubated with Fc receptor block (1:200, BD Bioscience) for 15 minutes at room temperature. Then, cells were stained with anti-mouse CD4-PE and and CD11c PE-Cy7 antibodies for 15 minutes at room temperature. Compensation beads, single stained CTV cells, and unstained cells were used as compensation controls. Fluorescence was measured using a LSRII flow cytometer (BD Bioscience) and data analysis was performed using the FlowJo Software v. 10.5.3 (FlowJo, LLC). Percent proliferation was calculated as [frequency of CTV^low^—CD4^+^ T cells]/[frequency of CD4^+^ T cells] x 100%. Gating strategy for proliferating CD4^+^ T cells is provided in the Supplementary Information.

For examining the presence of iNKT cells, CD4^+^ T cells from **R-P4** vaccinated or adjuvant-treated mice that were labeled with CellTrace Violet (ThermoFisher) were co- cultured with BMDC at a ratio of 1:10 DC:T cells for 5 days as described previously. Cells were subsequently stained with antibodies against CD3, CD4 and CD11c (ThermoFisher) as well as a PBS-57 loaded CD1d tetramer or mock loaded tetramer (NIH Tetramer Core Facility). PBS-57 is an analog of alpha-galactosylceramide (alpha-GalCer).

### Isolation and adoptive transfer of iNKT cells

iNKT cells were isolated from **R-P4** vaccinated or adjuvant control mice using methods described [100,101] with the following modifications. Briefly, two weeks post immunization, mice were euthanized, and livers were perfused with 1X Liver Perfusion Medium (Gibco) for 4 mins followed by digestion with 0.5mg/ml Collagenase A in HBSS + 5mM HEPES for 4 mins. Livers were harvested and strained through a 100μM filter and cells were washed and resuspended in Hepatocyte Wash Medium (Gibco). Liver cell suspensions were centrifuged at 50 x g for 5 mins and the supernatant was aspirated and the process was repeated. Liver iNKT cells were enriched by overlaying 80% Percoll with liver cells resuspended in 40% Percoll and centrifuging at 1783 x g for 25 mins. Liver cells were counted and resuspended in EasySep media (2% FBS in PBS) at 1 x 10^8^ cells/ml. Isolation of TCRbeta^+^ cells from the liver was performed using the EasySep Release Mouse Biotin Positive Selection Kit (17655, StemCell) and biotinylated anti-TCRbeta (109204, Biolegend). Cell purities were greater than 99% (liver TCRbeta^+^) and included 30–50% iNKT cells as determined by PBS-57-loaded tetramer staining. The TCRbeta^+^ cells were resuspended in PBS and 1–3 x 10^6^ cells/mouse were adoptively transferred to recipient mice via the retro-orbital route. One day post-adoptive transfer, recipient mice were infected with HH WT GBS as described earlier. Mice were monitored for signs of morbidity and were euthanized at 24 hrs post infection or earlier if signs of morbidity were observed. Peritoneal fluid, spleen, lung and brain were harvested, homogenized in PBS and serially diluted and plated on TSA for GBS enumeration.

### Analysis of iNKT cell responses *in vitro*

TCRbeta^+^ cells were isolated from livers of **R-P4** vaccinated and adjuvant control mice. Cells were co-cultured with bone marrow-derived Dendritic cells (DCs) that had been generated with GM-CSF and pulsed with either 5 μM **R-P4** or media or UV-treated WT GBS NCTC 10/84 or isogenic UV-treated GBS NCTC 10/84 ΔcylE (1 x 10^6^ CFU) for 24 hours prior to co-culture. Ratio of DC:T cells was 1:1–1:3. Controls included pulsed DCs alone. After 5 days of co-culture at 37°C with 5% CO_2_, supernatants were harvested, and cytokine concentrations were determined using the Th1/Th2/Th9/Th17/Th22/Treg Cytokine 17 Plex Mouse ProcartaPlex Multiplex Panel (EPX170-26087-901, ThermoFisher).

### Hemolysis inhibition assays

Purified granadaene was diluted in PBS to 1.25μM and treated with serum (1:1000 dilution) from **R-P4** or adjuvant treated WT, CD1d^-/-^ or Traj18^-/-^ mice in a final volume of 100μL. These samples were incubated for 1 hour at room temperature and protected from light. EDTA-treated human red blood cells (RBC) were diluted in PBS to make a 1% RBC solution. Granadaene-treated samples were overlayed with 100μL of 1% RBC solution and incubated for 1 hour at 37°C. After the incubation, samples were centrifuged for 8 min at 1,000 x g to pellet unlysed RBCs. Supernatants were assessed for hemoglobin release by measuring absorbance at 420nm. RBCs treated with 0.1% Triton X-100 and RBCs only samples were included as positive and negative control for hemolysis, respectively. % hemolysis was calculated relative to positive and negative control.

### Ascending GBS infection

Female mice were immunized with **R-P4** or adjuvant as indicated above. Four days following their booster dose, mice underwent timed pairing with isogenic males for 48 hours. Female mice were monitored for signs of pregnancy including weight gain and palpation to detect pups. Pregnant **R-P4** or adjuvant mice were infected intravaginally with 1 × 10^8^ CFU GBS (strain NCTC10/84) using a micropipette tip (P10; Rainin) on E15 using methods described [98]. Infected mice were monitored twice daily for signs of preterm labor (vaginal bleeding or pups in cage). At 72 hours post-infection or at the onset of preterm labor, dams were euthanized and pup viability was noted. Maternal blood, lower genital tract, uterus, and spleen were collected. The 2 left and right proximal and distal pups and placentas were also collected. Organs were homogenized, serially diluted and plated to enumerate GBS CFU.

### Statistical analysis

A p-value < 0.05 was considered significant. Statistical tests for each experiment are mentioned in the respective figure legends. Unless otherwise noted, an unpaired t test or one-way ANOVA with Tukey’s post-test was used to compare groups in the *in vitro* assays. All *in vitro* experiments were performed three independent times in technical triplicate (unless otherwise noted). CFU comparison between treatment groups in the *in vivo* experiments were determined using a Mann-Whitney test or Kruskal-Wallis test with Dunn’s test. GraphPad Prism (version 8.2.1) was used to compute all statistical tests.

## Supporting information

S1 Fig Gating strategy for T cell proliferation. The sequential gating strategy for T cell proliferation is shown from left to right with sample data from T cells isolated from an adjuvant immunized WT mouse, co-cultured with R-P4 pulsed WT DCs. Light scatter was used to include events based on size. Proliferating T cells were gated on CD4+/CellTrace violet (CTV)- population. Gates for CD4+ and CVT were defined using fluorescence minus one controls.
(TIF)

S2 Fig *Ex vivo* proliferation of T cells (WT, CD1d^-/-^, or Traj18^-/-^) in response to R-P4 stimulation by DCs (WT, CD1d^-/-^). (A, B) WT DCs were pulsed with R-P4 and co-cultured with CellTrace Violet (CTV) labeled T cells isolated from R-P4 vaccinated (A) or adjuvant control (B) mice that were WT, CD1d^-/-^, or Traj18^-/-^. After 5–7 days of co-culture, proliferating CD4+ T cells were identified by FACS staining. Proliferation is expressed as a percentage of CD4+ cells that have divided. Representative graph from 3 separate experiments is shown (C) CD1^-/-^ DCs were pulsed with R-P4 and co-cultured with CellTrace Violet (CTV) labeled T cells isolated from R-P4 vaccinated or adjuvant control WT mice. After 5–7 days of co-culture, proliferating CD4+ T cells were identified by FACS staining as above.
(TIF)

S3 Fig Proliferating T cells in response to R-P4 stimulation by DCs include iNKT cells. (A) Proliferating T cells from R-P4 vaccinated WT mice were gated based on Forward vs Side Scatter. Within this subset, CD3+ T cells were gated based on CD3 positivity and CD11c negativity. Subsequently CD3+CD4+ T cells were gated based on CD4 positivity. (B) PBS-57 (alpha-GalCer):CD1d Tetramer expression was then determined by gating CD3+CD4+ T cells that were Tetramer positive but negative for a mock-loaded A tetramer control.
(TIF)

S4 Fig Gating strategy for liver iNKT cells. (A, B) Single cells isolated R-P4 vaccinated WT mice were gated based on Forward vs Side Scatter. TCRb+ T cells were gated based on TCRb positivity and PBS-57 (a-GalCer):CD1d Tetramer expression was determined.
(TIF)

S5 Fig ^1^H-NMR spectra of two different batches of R-P4. (TIF)

S1 Text Chemical synthesis of R-P4. (DOCX)

S1 Data Source data for figures. (XLSX)
