# Supplementary figures and images for: CD1 and iNKT cells mediate immune responses against the GBS hemolytic lipid toxin induced by a non-toxic analog
Source: PLoS Pathog. 2023 Jun 29;19(6):e1011490. doi: 10.1371/journal.ppat.1011490 (PMC10337943; doi:10.1371/journal.ppat.1011490)

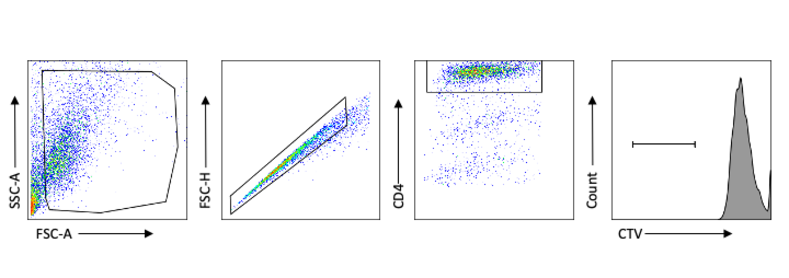

Supplement: S1 Fig — The sequential gating strategy for T cell proliferation is shown from left to right with sample data from T cells isolated from an adjuvant immunized WT mouse, co-cultured with R-P4 pulsed WT DCs. Light scatter was used to include events based on size. Proliferating T cells were gated on CD4+/CellTrace violet (CTV)- population. Gates for CD4+ and CVT were defined using fluorescence minus one controls. (TIF) [file ppat.1011490.s001.tif]

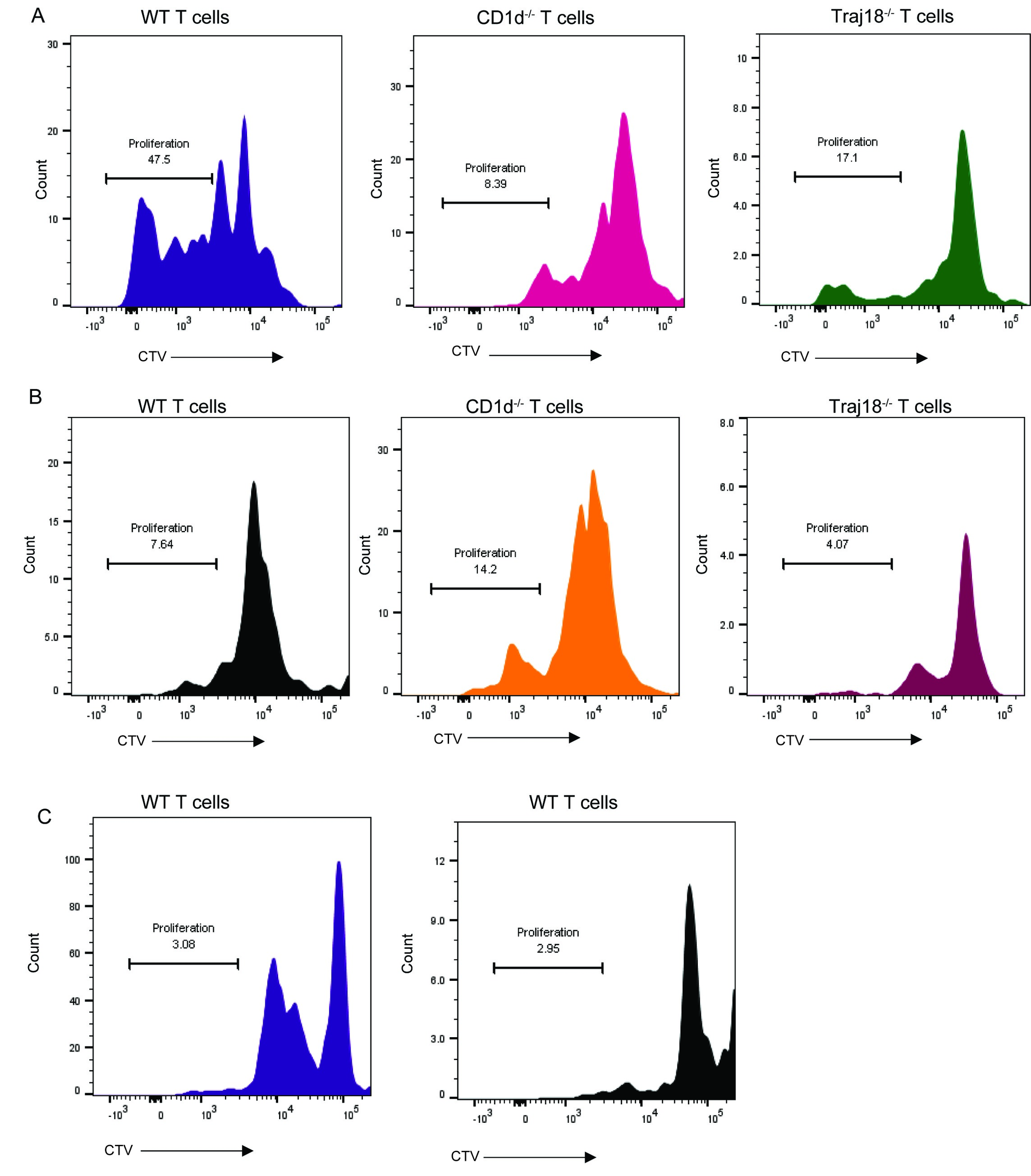

Supplement: S2 Fig — (A, B) WT DCs were pulsed with R-P4 and co-cultured with CellTrace Violet (CTV) labeled T cells isolated from R-P4 vaccinated (A) or adjuvant control (B) mice that were WT, CD1d-/-, or Traj18-/-. After 5–7 days of co-culture, proliferating CD4+ T cells were identified by FACS staining. Proliferation is expressed as a percentage of CD4+ cells that have divided. Representative graph from 3 separate experiments is shown (C) CD1-/- DCs were pulsed with R-P4 and co-cultured with CellTrace Violet (CTV) labeled T cells isolated from R-P4 vaccinated or adjuvant control WT mice. After 5–7 days of co-culture, proliferating CD4+ T cells were identified by FACS staining as above. (TIF) [file ppat.1011490.s002.tif]

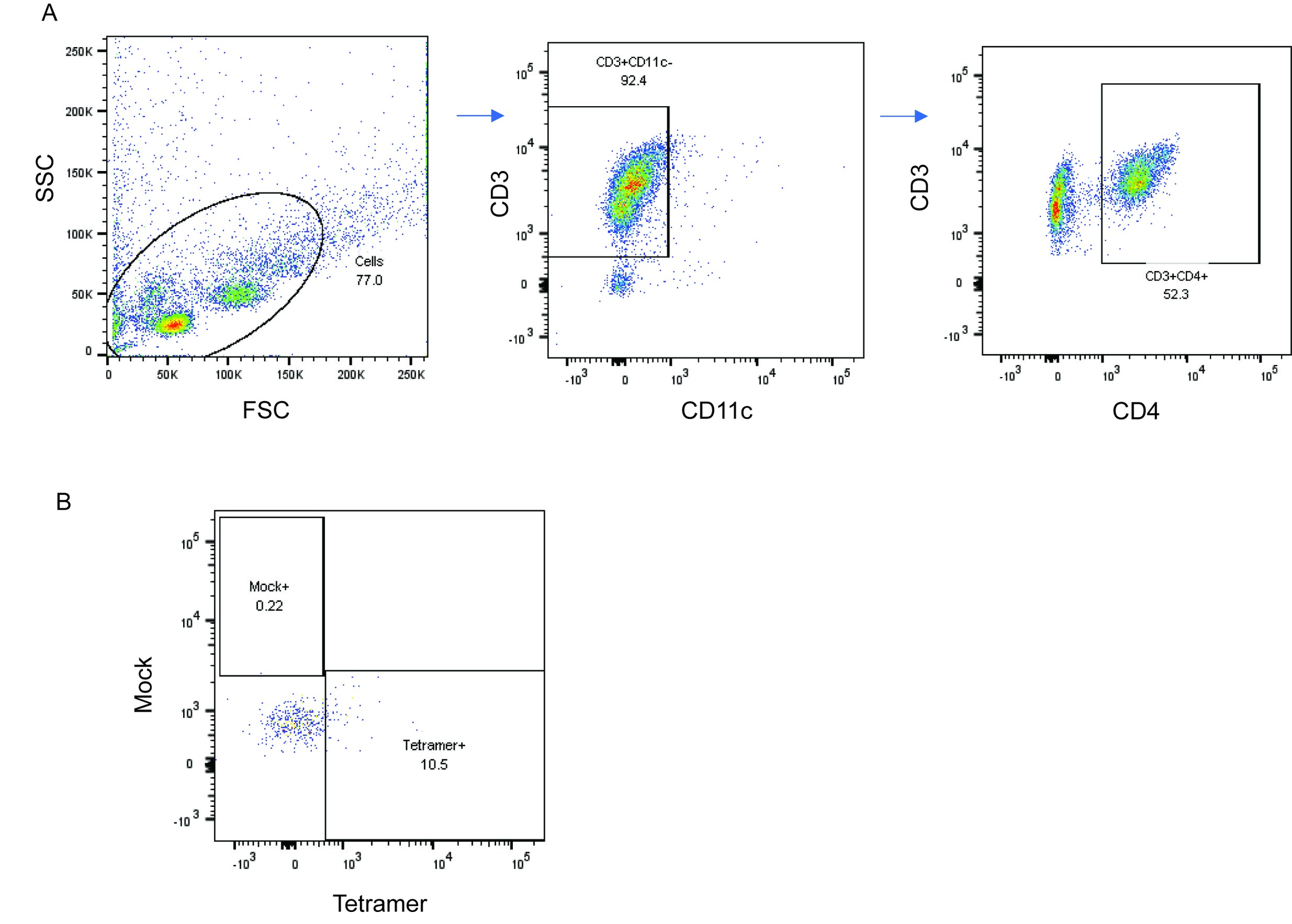

Supplement: S3 Fig — (A) Proliferating T cells from R-P4 vaccinated WT mice were gated based on Forward vs Side Scatter. Within this subset, CD3+ T cells were gated based on CD3 positivity and CD11c negativity. Subsequently CD3+CD4+ T cells were gated based on CD4 positivity. (B) PBS-57 (alpha-GalCer):CD1d Tetramer expression was then determined by gating CD3+CD4+ T cells that were Tetramer positive but negative for a mock-loaded A tetramer control. (TIF) [file ppat.1011490.s003.tif]

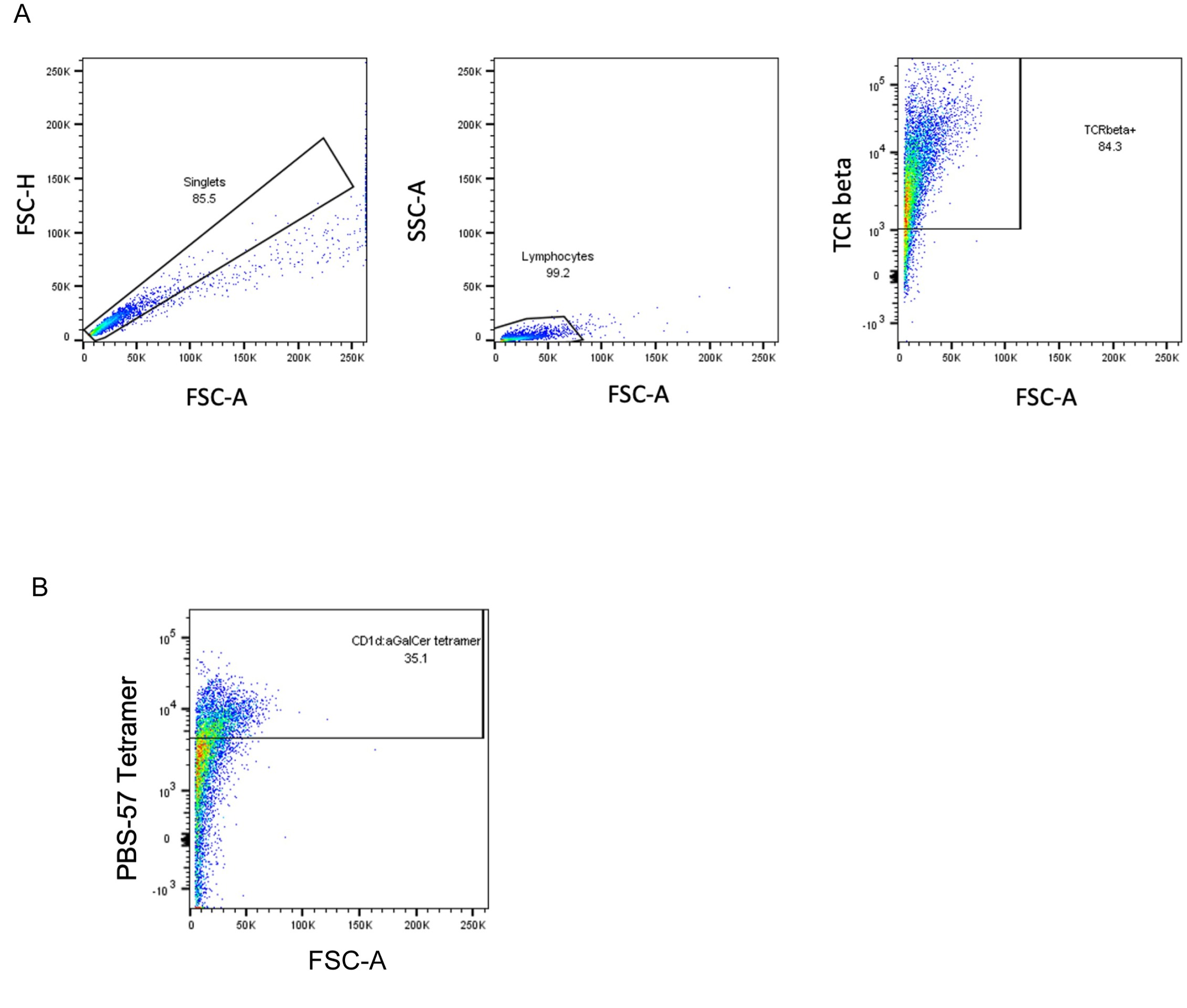

Supplement: S4 Fig — (A, B) Single cells isolated R-P4 vaccinated WT mice were gated based on Forward vs Side Scatter. TCRb+ T cells were gated based on TCRb positivity and PBS-57 (a-GalCer):CD1d Tetramer expression was determined. (TIF) [file ppat.1011490.s004.tif]

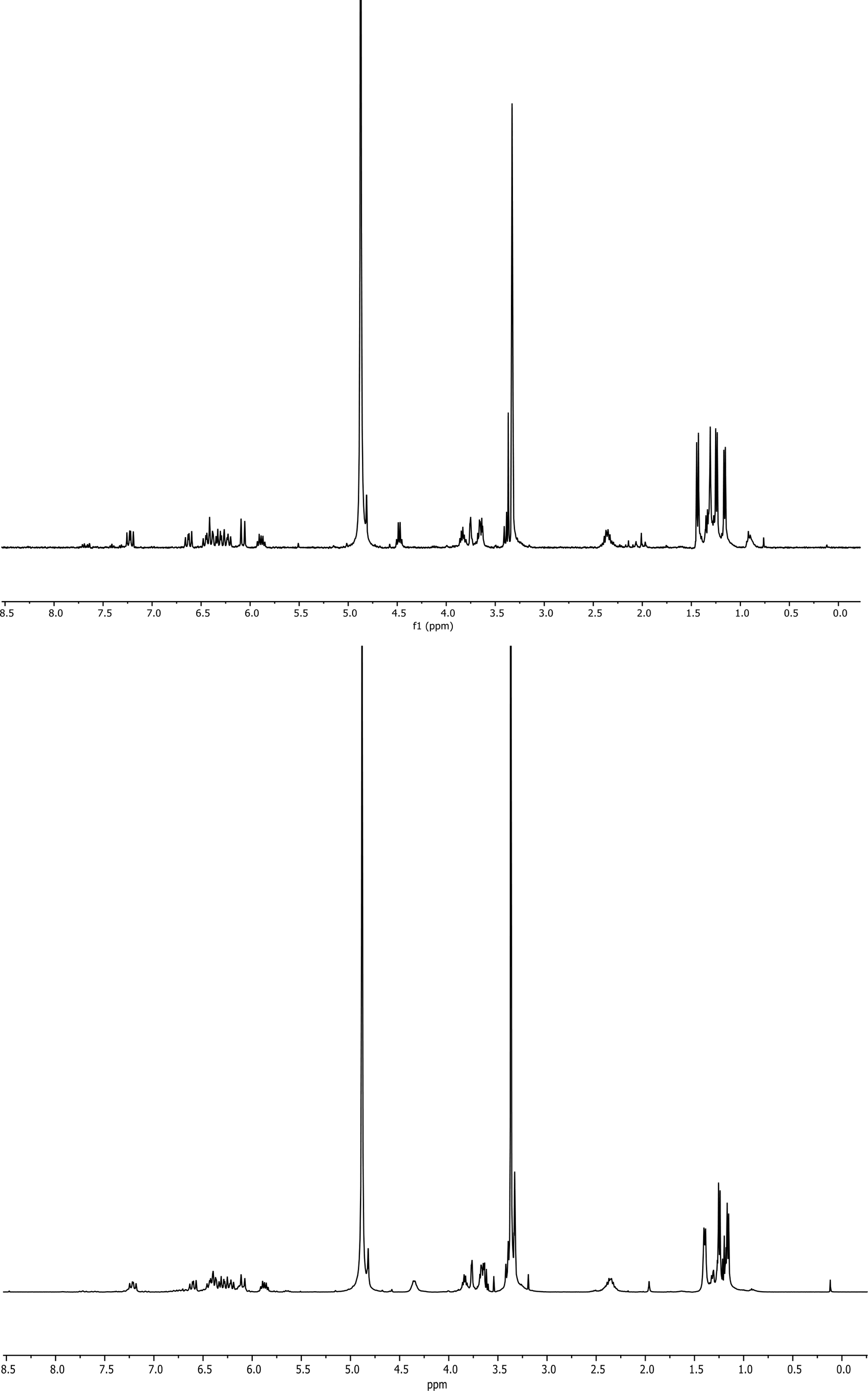

Supplement: S5 Fig — (TIF) [file ppat.1011490.s005.tif]
