# Supplementary material for: CD1 and iNKT cells mediate immune responses against the GBS hemolytic lipid toxin induced by a non-toxic analog
Source: PLoS Pathog. 2023 Jun 29;19(6):e1011490. doi: 10.1371/journal.ppat.1011490 (PMC10337943; doi:10.1371/journal.ppat.1011490)
Supplement: S1 Text — (DOCX) [file ppat.1011490.s006.docx]

### Chemical Synthesis of R-P4

Synthesis of L-alanine derivative **3**

Scheme 1. Synthesis of compound **3**

Synthesis of α-bromoamide **2**: To a solution of L-alanine (1.2 g, 8.6 mmol) in CH_2_Cl_2_ (50 mL), Et_3_N (2.61 g, 25.8 mmol), and bromoacetyl chloride (2.03 g, 12.9 mmol) were added at 0 ºC. The mixture was stirred at room temperature for 2 h. Then, EtOAc was added, and the mixture was washed with 10% aqueous HCl solution, sat. aq. NaHCO_3_, and brine. The organic layer was dried over anhyd. Na_2_SO_4_, and the solvent removed. The residue was purified by flash chromatography (EtOAc/hexane 3:7) to yield bromoamide **2** (0.99 g, 51%). Its ^1^H and ^13^C NMR spectra matched with those previously described (124).

Preparation of phosphonate **3**: To a sample of bromoamide **2** (0.99 g, 4.4 mmol), P(OEt)_3_ (0.81 g, 4.9 mmol) was added, and the mixture was stirred at 100 ºC for 12 h. Phosphonate **3** was obtained without further purification (1.05 g, 86%). Brown oil. ^1^H NMR (400 MHz, CDCl_3_) δ 7.22 (d, *J* = 7.3 Hz, 1H), 4.56 (quint, *J* = 7.2 Hz, 1H), 4.21–4.10 (m, 4H), 3.73 (s, 3H), 2.87 (d, *^2^J*_H-P_ = 20.7 Hz, 2H), 1.41 (d, *J* = 7.2 Hz, 3H), 1.33 (td, *^3^J*_H-H_ = 7.1; *^3^J*_H-P_ = 1.6 Hz, 6H). ^13^C NMR (101 MHz, CDCl_3_) δ 172.9 (C), 163.7 (d, ^2^*J*_C-P_ = 3.9 Hz, C), 62.9 (d, *^2^J*_C-P_ = 6.1 Hz, CH2), 62.8 (d, *^2^J*_C-P_ = 6.2 Hz, CH_2_), 52.4 (CH_3_), 48.4 (CH), 35.1 (d, *^1^J*_C-P_ = 130.9 Hz, CH_2_), 18.0 (CH_3_), 16.3 (d, *^3^J*_C-P_ = 6.0 Hz, CH_3_). ^31^P NMR (202 MHz, CDCl_3_) δ 22.25. HRMS (ESI): [M+Na]^+^ calcd. C_10_H_20_NPO_6_Na: 304.0920, found: 304.0928.

Synthesis of **R-P4**

Scheme 2. Synthesis of **R-P4**

General protocol for Horner–Wadsworth–Emmonds reaction (GP1): A mixture of the corresponding phosphonate (2.5 equiv.), and NaH (2.5 equiv., 60% purity) in THF (0.25 M) was stirred at 0 °C for 10 min Then, the corresponding aldehyde (1 equiv.) was added dropwise for 10 min, and the mixture was stirred at room temperature for 0.5–1.5 h. Then, saturated aqueous NH4Cl was added, and THF was removed under reduced pressure. The residue was solved in EtOAc, and the mixture was washed with saturated aqueous NH_4_Cl and brine, dried over anhydrous Na_2_SO_4_, and the solvent was removed. Products were purified by flash chromatography on silica gel (EtOAc/hexane mixtures) and characterized by ^1^H- and ^13^C-NMR and HRMS.

Synthesis of aldehyde **6**: To a solution of ester **5** (500 mg, 2.00 mmol) in Et_2_O (2 mL), DIBAL-H (2.2 mL, 2.2 mmol, 1 M in THF) was added, and the mixture was stirred at –78 ºC for 40 min. Then, the reaction was quenched with H_2_O, diluted with EtOAc, and washed with 10% aqueous HCl solution, and brine. The mixture was dried over anhyd. Na_2_SO_4_ and the solvent removed. The residue was purified by flash chromatography (EtOAc/hexane 1:9) to yield **6** (400 mg, 97%). Its ^1^H and ^13^C NMR spectra matched with those previously described. (2)

Synthesis of ester **7**: According to GP1, ester **7** was synthesized from aldehyde **6** in 75% yield. Colorless oil; $[{\alpha]}_{D}^{25}$ = + 2.6 (c = 0.01, CHCl_3_). Its ^1^H and ^13^C NMR spectra matched with previously described (125).

Synthesis of alcohol **8**: Following the procedure used for the synthesis of **6**, alcohol **8** was prepared from ester **7** in 97% yield (EtOAc/hexane 1.5:8.5). Light yellow oil; $[{\alpha]}_{D}^{25}$ = +5.6 (c = 0.01, CHCl_3_). Its ^1^H and ^13^C NMR spectra matched with those previously described. (2)

Major *E,E,E*-isomer, ^1^H NMR (500 MHz, CDCl_3_) δ 6.27 (ddt, *J* = 15.2, 10.2, 1.4 Hz, 1H, 3-CH), 6.20 (dd, *J* = 14.8, 10.2 Hz, 1H, 4-CH), 6.13 (dd, *J* = 14.9, 10.4 Hz, 1H, 5-CH), 6.07 (ddt, *J* = 15.0, 10.2, 1.4 Hz, 1H, 6-CH), 5.82 (dt, *J* = 15.2, 6.0 Hz, 1H, 2-CH), 5.71 (dt, *J* = 15.0, 7.5 Hz, 1H, 7-CH), 4.19 (t, *J* = 5.2 Hz, 2H, 1-CH_2_), 3.83 (h, *J* = 6.0 Hz, 1H, 9-CH), 2.29–2.15 (m, 2H, 8-CH_2_), 1.13 (d, *J* = 6.1 Hz, 3H, 10-CH_3_), 0.88 (s, 9H, OSi(CH_3_)_2_C(C*H*_3_)_3_), 0.04 (s, 3H, OSi(C*H*_3_)_2_C(CH_3_)_3_), 0.03 (s, 3H, OSi(C*H*_3_)_2_C(CH_3_)_3_). ^13^C NMR (126 MHz, CDCl_3_) δ 133.7 (4-CH), 132.5 (7-CH), 132.3 (6-CH), 132.0 (3-CH), 131.4 (2-CH), 130.0 (5-CH), 68.7 (9-CH), 63.7 (1-CH_2_), 43.3 (8-CH_2_), 26.0 (CH_3_, OSi(*C*H_3_)_2_C(CH_3_)_3_), 23.7 (10-CH_3_), 18.3 (C, OSi(CH_3_)_2_*C*(CH_3_)_3_), –4.4 (CH_3_, OSi(*C*H_3_)_2_C(CH_3_)_3_), –4.5 (CH_3_, OSi(*C*H_3_)_2_C(CH_3_)_3_). HRMS (ESI): [M]^+^ calcd. C_16_H_30_O_2_Si: 282.2015, found: 282.2026.

Synthesis of aldehyde **9**: To a solution of the corresponding alcohol **8** (1 mmol) in CH_2_Cl_2_ (0.3 M) at 0 °C, Dess–Martin periodinane (1.5 mmol) was added, and the mixture was stirred at room temperature for 2 h. Then, the solvent was removed, and EtOAc was added. The organic layer was washed with a 1:1 solution of saturated NaHCO_3_ and 10% Na_2_S_2_O_3_, dried over anhydrous Na_2_SO_4_, and the solvent removed. The crude was purified by flash chromatography on silica gel (EtOAc/hexane 0.5:9.5) to give aldehyde **9** in 64% yield. Deep yellow oil; $[{\alpha]}_{D}^{25}$ = +9.1 (c = 0.01, CHCl_3_); Spectroscopic data matched with those previously described. (2) *E,E,E*-isomer: ^1^H NMR (400 MHz, CDCl_3_) δ 9.55 (d, *J* = 7.9 Hz, 1H), 7.12 (dd, *J* = 15.3, 11.1 Hz, 1H), 6.65 (dd, *J* = 14.9, 10.6 Hz, 1H), 6.36 (dd, *J* = 14.9, 11.1 Hz, 1H), 6.24–6.10 (m, 2H), 6.03 (dt, *J* = 15.0, 7.4 Hz, 1H), 3.89 (h, *J* = 6.1 Hz, 1H), 2.32–2.26 (m, 2H), 1.15 (d, *J* = 6.1 Hz, 3H), 0.88 (s, 9H), 0.05 (s, 3H), 0.04 (s, 3H). ^13^C NMR (101 MHz, CDCl_3_) δ 193.7 (CH), 152.4 (CH), 143.1 (CH), 139.0 (CH), 131.9 (CH), 131.0 (CH), 128.3 (CH), 68.4 (CH), 43.5 (CH_2_), 26.0 (CH_3_), 23.9 (CH_3_), 18.3 (C), –4.3 (CH_3_), –4.6 (CH_3_). HRMS (ESI): [M+Na]^+^ calcd. C_16_H_28_O_2_SiNa: 303.1750, found: 303.1752.

Synthesis of ester **10**: According to GP-1, ester **10** was synthesized from aldehyde **9** in 90% yield (EtOAc/hexane 2:8). Light brown oil; $[{\alpha]}_{D}^{25}$ = +2.1 (c = 0.01, CHCl_3_); Spectroscopic data matched with those previously described. (2) Major *E,E,E,E* isomer: ^1^H NMR (500 MHz, CDCl_3_) δ 7.27 (dd, *J* = 14.9, 11.3 Hz, 1H), 6.55 (dd, *J* = 14.8, 11.0 Hz, 1H), 6.36 (dd, *J* = 14.9, 10.7 Hz, 1H), 6.27 (dd, *J* = 14.8, 11.3 Hz, 1H), 6.19 (dd, *J* = 14.9, 11.0 Hz, 1H), 6.12 (dd, *J* = 15.1, 10.7 Hz, 1H), 6.02 (d, *J* = 7.5 Hz, 1H), 5.85 (d, *J* = 14.9 Hz, 1H), 5.83–5.78 (m, 1H), 4.70 (quint, *J* = 7.2 Hz, 1H), 3.85 (h, *J* = 6.0 Hz, 1H), 3.76 (s, 3H), 2.30–2.18 (m, 2H), 1.44 (d, *J* = 7.1 Hz, 3H), 1.13 (d, *J* = 6.1 Hz, 3H), 0.88 (s, 9H), 0.04 (s, 3H), 0.03 (s, 3H). ^13^C NMR (126 MHz, CDCl_3_) δ 173.8 (C), 165.6 (C), 141.8 (CH), 140.4 (CH), 137.0 (CH), 134.7 (CH), 132.4 (CH), 130.3 (CH), 129.4 (CH), 122.3 (CH), 68.6 (CH), 52.7 (CH_3_), 48.2 (CH), 43.5 (CH_2_), 26.0 (CH_3_), 23.8 (CH_3_), 18.9 (CH_3_), 18.3 (C), –4.4 (CH_3_), –4.5 (CH_3_). HRMS (ESI): [M+Na]^+^ calcd. C_22_H_37_NO_4_SiNa: 430.2384, found: 430.2399.

Synthesis of alcohol **11**: To a solution of ester **10** (292 mg, 0.717 mmol) in MeOH (2 mL), amberlyst 15(H) was added (up to pH 5), and the mixture was stirred at room temperature for 90 min. Then, the residue was purified by flash chromatography (MeOH/CH_2_Cl_2_ 1:9) to yield **11** (110 mg, 52%). Light yellow oil; $[{\alpha]}_{D}^{25}$ = –5.3 (c = 0.09, CHCl_3_); Spectroscopic data matched with those previously described. (2) Major *E,E,E,E* isomer: ^1^H NMR (500 MHz, CDCl_3_) δ 7.27 (dd, *J* = 14.9, 11.3 Hz, 1H), 6.54 (dd, *J* = 14.8, 11.0 Hz, 1H), 6.37 (dd, *J* = 15.0, 10.5 Hz, 1H), 6.29 (dd, *J* = 14.8, 11.4 Hz, 1H), 6.26–6.17 (m, 2H), 6.04 (d, *J* = 7.4 Hz, 1H), 5.87 (d, *J* = 15.1 Hz, 1H), 5.84–5.79 (m, 1H), 4.70 (quint, *J* = 7.2 Hz, 1H), 3.88 (h, *J* = 6.4 Hz, 1H), 3.76 (s, 3H), 2.36–2.22 (m, 2H), 1.44 (d, *J* = 7.2 Hz, 3H), 1.21 (d, *J* = 6.2 Hz, 3H). ^13^C NMR (126 MHz, CDCl_3_) δ 173.8 (C), 165.6 (C), 141.7 (CH), 140.1 (CH), 136.4 (CH), 133.5 (CH), 133.1 (CH), 131.0 (CH), 129.9 (CH), 122.6 (CH), 67.5 (CH), 52.7 (CH_3_), 48.3 (CH), 43.0 (CH_2_), 23.1 (CH_3_), 18.9 (CH_3_). HRMS (ESI): [M+Na]^+^ calcd. C_16_H_23_NO_4_Na: 316.1519, found: 316.1507.

Synthesis of rhamnose derivative **12**: To a deoxygenated solution of alcohol **11** (114 mg, 0.389 mmol) and anhydrous Hg(CN)_2_ (98 mg, 0.39 mmol) in dry CH_3_CN (2 mL), bromorhamnose (206 mg, 0.585 mmol) was added and the mixture was stirred at 40 °C until completion (~3 h). Then, the solvent was removed. The residue was solved in EtOAc and washed with 1M solution of KBr, saturated solution of NaHCO_3_ and water. The organic layer was dried over anhyd Na_2_SO_4_ and the solvent was removed. The residue was submitted to flash chromatography (EtOAc/hexane 3:7) to yield ester **12** (121 mg, 55%). Brown oil; $[{\alpha]}_{D}^{25}$ = -36.6 (c = 0.01, CHCl3); Spectroscopic data matched with those previously described. (2) Major *E,E,E,E* isomer: ^1^H NMR (500 MHz, CDCl_3_) δ 7.26 (dd, *J* = 14.9, 11.3 Hz, 1H), 6.54 (dd, *J* = 14.4, 11.2 Hz, 1H), 6.40–6.14 (m, 4H), 6.05 (d, *J* = 7.4 Hz, 1H), 5.87 (d, *J* = 14.9 Hz, 1H), 5.79 (dt, *J* = 15.4, 7.6 Hz, 1H), 5.27 (dd, *J* = 10.2, 3.6 Hz, 1H), 5.17 (bs, 1H), 5.04 (t, *J* = 10.2 Hz, 1H), 4.83 (d, *J* = 1.8 Hz, 1H), 4.75–4.65 (quint, *J* = 7.2 Hz, 1H), 4.02–3.86 (m, 1H), 3.83–3.78 (m, 1H), 3.76 (s, 3H), 2.44–2.36 (m, 1H), 2.34–2.27 (m, 1H), 2.14 (s, 3H), 2.01 (s, 3H), 1.98 (s, 3H), 1.44 (d, *J* = 7.2 Hz, 3H), 1.17 (d, *J* = 6.3 Hz, 3H), 1.15 (d, *J* = 6.2 Hz, 3H). ^13^C NMR (126 MHz, CDCl_3_) δ 173.7 (C), 170.2 (C), 170.0 (C), 165.4 (C), 141.5 (CH), 140.0 (CH), 136.3 (CH), 133.1 (CH), 132.9 (CH), 130.7 (CH), 129.6 (CH), 122.4 (CH), 95.1 (CH, *^1^J*_C-H_ = 170 Hz), 72.8 (CH), 71.1 (CH), 70.5 (CH), 69.2 (CH), 66.5 (CH), 52.5 (CH_3_), 48.1 (CH), 40.4 (CH_2_), 20.9 (CH_3_), 20.8 (CH_3_), 20.7 (CH_3_), 18.9 (CH_3_), 18.7 (CH_3_), 17.3 (CH_3_). HRMS (ESI): [M+H]^+^ calcd C_28_H_40_NO_11_: 566.2595, found: 566.2607.

Preparation of **R-P4**: To a solution of ester-**12** (27 mg, 0.05 mmol) in MeOH (1 mL), KOH 2M aqueous solution (0.29 mL, 0.57 mmol) was added and the mixture was stirred for 5 h. Then, washed amberlyst was added until pH 5, the mixture was submitted to flash chromatography (MeOH/CH_2_Cl_2_ 1:1) to yield **R-P4** (15 mg, 76%). Light brown oil;$[{\alpha]}_{D}^{25}$ = –16.4 (c = 0.04, CHCl_3_); Mixture of isomers. Spectroscopic data matched with those previously described. (2) Data of major *E,E,E,E* isomer are given: ^1^H NMR (500 MHz, CD_3_OD) δ 7.18 (dd, *J* = 15.1, 11.0 Hz, 1H), 6.59 (dd, *J* = 14.8, 10.9 Hz, 1H), 6.44–6.32 (m, 2H), 6.27 (dd, *J* = 14.9, 10.9 Hz, 1H), 6.21 (dd, *J* = 15.2, 10.6 Hz, 1H), 6.07 (d, *J* = 14.8 Hz, 1H), 5.86 (dt, *J* = 14.8, 7.2 Hz, 1H), 4.34 (q, *J* = 7.6 Hz, 1H), 3.83 (m, 1H), 3.74 (bs, 1H), 3.68–3.61 (m, 2H), 3.37 (t, *J* = 9.5 Hz, 2H), 2.45–2.28 (m, 2H), 1.38 (d, *J* = 7.1 Hz, 3H), 1.23 (d, *J* = 6.4 Hz, 3H), 1.15 (d, *J* = 6.1 Hz, 3H). ^13^C NMR (126 MHz, CD_3_OD) δ 179.4 (C), 168.0 (C), 141.7 (CH), 140.9 (CH), 137.6 (CH), 134.3 (CH), 134.0 (CH), 131.9 (CH), 131.1 (CH), 124.5 (CH), 99.3 (CH), 74.0 (CH), 73.4 (CH), 72.84 (CH), 72.4 (CH), 70.1 (CH), 51.6 (CH), 42.9 (CH_2_), 19.19 (CH_3_), 19.17 (CH_3_), 17.9 (CH_3_). HRMS (ESI): [M+Na]^+^ calcd. C_21_H_31_NO_8_Na: 448.1941, found: 448.1932. Solubility: MeOH, DMSO.
